# Breeding in bread-making wheat varieties for Mediterranean climate: the need to get resilient varieties

**DOI:** 10.3389/fnut.2024.1393076

**Published:** 2024-08-07

**Authors:** Benvindo Maçãs, Rita Costa, Conceição Gomes, Ana Sofia Bagulho, Nuno Pinheiro, José Moreira, Armindo Costa, Manuel Patanita, José Dores, Sara Rodrigo

**Affiliations:** ^1^Instituto Nacional de Investigação Agrária e Veterinária, Elvas, Portugal; ^2^GeoBiotec, Faculdade de Ciências e Tecnologia, Universidade Nova de Lisboa, Caparica, Portugal; ^3^Instituto Mediterrâneo para a Agricultura, Ambiente e Desenvolvimento, MED, Universidade de Évora, Evora, Portugal; ^4^Instituto Politécnico de Beja – Escola Superior Agrária, Beja, Portugal; ^5^Instituto de Investigación de la Dehesa (INDEHESA), Universidad de Extremadura, Badajoz, Spain

**Keywords:** germplasm, wheat breeding, quality, climate change, resilience

## Abstract

**Introduction:**

Being one of the “big three” most cultivated cereals in the world, wheat plays a crucial role in ensuring global food/nutrition security, supplying close to 20% of the global needs for calories and proteins. However, the increasingly large fluctuations between years in temperatures and precipitation due to climate change cause important variations in wheat production worldwide. This fact makes wheat breeding programs a tool that, far from going out of fashion, is becoming the most important solution to develop varieties that can provide humanity with the sufficient amount of food it demands without forgetting the objective of quality.

**Material and methods:**

The National Institute of Agricultural and Veterinary Research in Portugal has carried out a long-term experiment (9 years) in different locations to test four different bread-making wheat cultivars, each representing important variations in germplasm. Wheat yield and quality traits obtained by official methods were recorded in 18 different environments regarding temperature and precipitation.

**Results and discussion:**

According to the ANOVA and PCA, protein content, wet gluten, dough tenacity, and extensibility were found to be highly affected by the environment. Paiva cultivar presented a higher yield in almost all the tested environments, but its quality traits varied enormously. Contrary behavior was recorded for Valbona cultivar. Antequera cultivar, with a production ranging between 4.7 and 9.3 tons/ha and a protein content between 11 and 16.8%, seems to be the most resilient cultivar regarding both productivity and quality of the flour with reference to changes in the main climate traits. The most ancient cultivar, Roxo, released in 1996, showed the worst results in this experiment, supporting the need to continue working in wheat breeding to meet the unavoidable changes in the environment.

## 1 Introduction

For more than three quarters of a century, the Portuguese Unit of Research on Genetic Resources, Ecophysiology, and Plant Breeding of the National Institute of Agricultural and Veterinary Research (previously known as Plant Breeding Station) has been entering new wheat varieties in the National Catalog. To obtain new varieties, breeders start with a wide genetic variability and a well-designed program of artificial crossings between two wheat genotypes, subsequently exerting a broad selection pressure on future generations. Finally, a new homogeneous and stable cultivar with the searched characteristics is obtained. However, the big drawback of this process is the time spent on it, since it takes 10 or more generations before registration (1).

According to Fisher et al. (2), the principles of breeding are similar for many crops since they are cultivated in similar ways. In addition, all these new cultivars have to face analogous challenges, including (i) resisting or tolerating diseases and pests; and (ii) adapting to variable temperatures, water supply, light, and soil conditions. However, significant yield gaps in many annual crops attest to the importance of selecting appropriately for heritable traits through plant breeding (3). These traits include not only high yield and good commercial expectations (quality) but also resistance to biotic stresses (pests and diseases, mainly), as well as abiotic ones, within target environments. In this context, it is noteworthy that the varieties historically have been selected in a given environment or climate, but nowadays, under extreme and unpredictable weather conditions, in a scenario of increased temperatures in dry areas and a more erratic rainfall pattern, as stated in recent IPCC reports,^1^ resilience in new varieties to be adapted to different and extreme weather events is, more than desired, essential.

Wheat (*Triticum aestivum* L.) is among the world’s most important staple food crops (4), supplying a fifth of global food calories and proteins (4, 5). According to Erestein et al. (6), since 1961, the global area under wheat production has oscillated between 200 and 240 M ha, making wheat the most widely grown crop in the world. The world total wheat production in the last 5 years (with available data) ranged between 732 and 772 M tons; however, even when over 120 countries all along the five continents cultivate wheat, the major global contribution to this cereal production is done by Asia and Europe (44 and 34%, respectively), followed by the Americas (15%). China, India, Russia, the USA, and France are the top five world producers (7). Land use changes or decreases in the productivity (mainly due to weather events) of one of these producers could contribute to a lack in the global wheat supply; thus, the expansion of maize and soy bean in the Americas and the susceptibility of wheat to rising temperatures (8, 9) in the Great Plains of North America, which produces 30% of the world’s high-quality wheat exports (7), have been identified as critical threats (10).

Among the objectives of wheat breeding programs, it is possible to find a number of goals: rust’s resistance, due to the great importance of this disease worldwide (11–14), tolerance to drought (15, 16) or the development of new varieties suitable for organic production systems (17). All these objectives are related to yield: improve or, better said, avoid reducing the productivity of wheat varieties. However, quality in wheat is also important, and an emerging objective to take into account in wheat breeding programs. Thus, the reduction in gluten content to develop “celiac-safe” wheat (18) or the increase in the wheat plants’ ability to uptake and store in kernels essential minerals for humans such as Fe and Zn (19), together with kernel hardness, which determines the end-product quality (20), are new goals to achieve in concordance with the previous ones. In addition, it is still a challenge to solve the conflict between improving the yield or the grain protein concentration, due to the fact that there is usually a negative correlation between these parameters (21). This negative correlation is important because protein content, directly related to gluten yield, affects the quality of cereal-based products, including both the volume and appearance of baked goods and the brittleness of pasta. In this sense, both agricultural technologies applied during cropping and environmental conditions that occur during phenological development hugely influence these parameters (22).

Taking into account all the STATED above, it is necessary to clarify that well-funded crop breeding is important for the correct development of future agriculture and essential for global food security. Nowadays, private breeding plays a dominant role in high-profitability crops such as soya bean or corn, but other important crops (rice, wheat, pulses, etc.) are heavily dependent on public funding (23), and public research centers are responsible for maintaining genetic variability. However, private sector is getting more engaged in wheat breeding, especially in North America, increasing farmers and scientific concerns due to the differences in absolute and relative yields found between public and private experiment data. This fact was pointed out by Nti and Barkley (24), who finally attribute the differences to the different production practices and other specific environmental characteristics, but maybe it is something we should be cautious about.

The main concerns of any cereal breeding program are: ensuring global food/nutrition security for a growing world population; permanent climate change due to frost; high temperatures and changes in the overall rainfall patterns that could reduce yield stability and quality; and the occurrence of constraints caused by biotic stress (25). In this regard, testing modern and well-established wheat cultivars, focusing not only in yield but also in technological quality in different environments regarding rainfall patterns and temperatures, seems to be a good way to determine the resilience of the current cultivars against weather events. In this long-term experiment, the behavior of four cultivars representing common germplasm origin in different locations in the Mediterranean climate, with completely different rainfall patterns and temperatures, is to test their resilience to face climate change.

## 2 Materials and methods

### 2.1 Wheat germplasm

This study was performed with four cultivars of bread wheat (*Triticum aestivum* L.), namely Antequera, Paiva, Roxo, and Valbona. Paiva and Roxo are cultivars obtained from the Portuguese Wheat Breeding Program of the National Institute for Agrarian and Veterinarian Research (Elvas, Portugal), released in 2016 and 1995, respectively. Antequera and Valbona are commercial cultivars from Agrovegetal, Spain (2009), and Delley Semences et Plants, SA (2006), Italy, respectively. All are spring-type cultivars with early maturity, and, as indicated above, they represent important lines of breeding work, joining a representative germplasm by their origin.

### 2.2 Environmental conditions and field experiments

Field experiments were conducted during nine consecutive years, from 2015 to 2023 cropping seasons, in two different regions of Portugal: Elvas (Alto Alentejo region—AA) and Beja (Baixo Alentejo region—BA), representing the most important provinces in Portugal for bread wheat crops. Four different farms were used during the years of the experiment, and the combination of agronomic season (9 years) and region/farm (two per year) is considered as Environment (9 × 2 = 18). This means that in each region (AA and BA), one farm per year was used, all of them with similar soil conditions. The following tables show some important data about the studied environments (Env1–Env18) regarding sowing and harvesting data in any environment, as well as the altitude of the different farms used in the experiments over the years (Table 1), and the most important climatic conditions for the wheat crop in Mediterranean regions (Table 2), which are more detailed in Supplementary Figures S1–S18.

**Table 1:** Environment, altitude of the different farms used in the experiments, and sowing and harvesting dates of the trials.

Environment	Sowing date	Harvesting date	Geographical location
Env1	30 December 2014	02 July 2015	BA-1
Env2	09 December 2015	11 July 2016	BA-1
Env3	13 December 2016	21 June 2017	BA-2
Env4	07 December 2017	02 July 2018	BA-2
Env5	26 December 2018	03 July 2019	BA-2
Env6	13 December 2019	17 June 2020	BA-2
Env7	20 November 2020	07 July 2021	BA-2
Env8	03 December 2021	30 June 2022	BA-2
Env9	30 December 2022	06 July 2023	BA-2
Env10	16 December 2014	24 June 2015	AA-1
Env11	11 December 2015	06 July 2016	AA-1
Env12	28 December 2016	04 July 2017	AA-1
Env13	19 December 2017	09 July 2018	AA-1
Env14	06 December 2018	13 June 2019	AA-2
Env15	11 December 2019	23 June 2020	AA-2
Env16	06 January 2021	24 June 2021	AA-2
Env17	13 December 2021	14 June 2022	AA-2
Env18	25 January 2023	20 June 2023	AA-2

BA-1: Baixo Alentejo, Beja, Herdade do Outeiro (38°02′42″N, 8°15′59″O, 318 m.a.s.l.), BA-2: Baixo Alentejo, Beja, Quinta da Saúde (38°02′06, 44″N, 7°53′08, 55″O, 314 m.a.s.l), AA-1: Alto Alentejo, Elvas, Herdade da Comenda (38°53′39″N, 7°03′20″O, 214 m.a.s.l.), AA-2: Alto Alentejo, Elvas, Estaçao Nacional de Melhoramento de Plantas (38°53′19, 60″ N, 7°08′38, 53″O, 218 m.a.s.l.).

**Table 2:** Climatic data (minimum, maximum, and average temperature and total rainfall) in winter and spring from the 18 different environments.

	Winter (December–February)				Spring (March–May)				Total rainfall year (mm)
	Rainfall (mm)	Min. Temp. (°C)	Max. Temp. (°C)	Averg. Temp. (°C)	Rainfall (mm)	Min. Temp. (°C)	Max. Temp. (°C)	Averg. Temp. (°C)	
Environment 1	52.5	−0.6	20.1	9.7	82.6	3.1	38.7	17.1	416
Environment 2	158.4	9.8	14.6	12.2	171.9	2.4	33	14.2	461
Environment 3	160.1	−2	22.9	11.3	105.7	1.9	36.6	16.8	378
Environment 4	117.5	−0.3	20.9	10.3	387.5	2.9	30	14.2	604
Environment 5	85.3	−2.2	23.6	11.3	80.3	2.6	37	16.3	339
Environment 6	158.7	1.5	25.3	12.5	160.1	3.1	35.2	16.4	435
Environment 7	238.8	−2.7	23	11.1	70.3	3.2	34.1	16.3	529
Environment 8	82.5	1.1	25.7	12.5	172.7	2.3	35.7	16.3	366
Environment 9	177.7	−1.6	23.6	12.1	40.1	0	37.2	17.9	377
Environment 10	61.9	−3.8	21.8	8.9	130.9	1.9	38.3	17.4	505
Environment 11	140.4	−2.2	21.8	11.3	229.5	−0.1	32.8	14.2	610
Environment 12	156.9	−3.5	20.2	10.2	89.2	0.6	35.7	16.8	442
Environment 13	130.9	−2.7	20.4	9.5	382.8	0.8	30.2	14.6	591
Environment 14	63.1	−2.3	24.1	9.9	85.7	2.7	35	16.4	351
Environment 15	142.6	−0.8	24.1	11.5	277.6	1.3	34.9	16.2	556
Environment 16	225.9	−4.5	21	10.1	111.6	2	34.4	16.1	604
Environment 17	40.4	−1.1	23.2	10.9	125.1	0.7	36.2	16.1	279
Environment 18	320.8	−2.8	20.6	10.8	48.5	−3.5	35.3	17.5	574

Soils from both locations did not differ enormously, with all of them being classified as vertisols according to the Food and Agriculture Organization (FAO).^2^ All the studied soils showed a pH ranging between 6.8 and 8.1, with low organic matter in the soil (1.4–1.7%) and high contents of P (>200 ppm P_2_O_5_), Ca (2,400–3,500 ppm Ca), as well as low N content (16.38–19.89 kg N/ha). Regarding other relevant parameters, medium and high cation exchange capacity (CEC) and K (between 16 and 22 cmol kg^−1^ for CEC and 68 and 168 ppm for K_2_O) were found in the soils. Fertilizer supply was done according to soil analysis, as stated in the following paragraphs.

### 2.3 Experimental design

The experimental design was a randomized complete block design with three replications using a split-plot treatment arrangement. Each small plot size area was 12 m^2^ (10 m long and six rows, 20 cm apart).

### 2.4 Crop management

Fertilization was conducted with nitrogen fertilization at sowing time (40–42 UN) and three top-dressed fertilizations (40 tillering–60 booting–40 UN heading/flowering) any year in any farm. Two weed control treatments (at pre-emergence and post-emergence) and two antifungal treatments [stem elongation (GS30–GS33) and booting (GS41–GS47) were applied per year]. Conventional tillage management included moldboard plowing and disk harrowing at the beginning of autumn and/or vibrating tine cultivation to prepare a proper seedbed before sowing. Experiments were sown in December/January (Table 1) at a seeding rate of 350 grains m^−2^.

### 2.5 Measurements

Wheat harvest took place in June/July (Table 1) using a 1.5 m wide Nurserymaster Elite Plot Combine (Wintersteiger, Austria), and grain yield was determined. Thousand kernel weight (TKW) was obtained using a grain counter (Pfeuffer) according to ISO 520:2010. Test weight and total N content (consequently, protein content of grain and flour) were determined using near-infrared equipment (Infratec™ 1241 Grain Analyzer, Foss) according to EN 15948:2020. Bread-making wheat grain was ground with a Laboratory Mill CD1 (Chopin, France) to obtain white flour to test dough quality. The deformation work (W), dough tenacity (P), and extensibility (L) of the wheat flour were determined using a Chopin Alveograph (Model Alveo PC, Chopin, France), according to the standard ISO 27971:2023. Wet gluten content and falling number were performed in the flours according to the standards ISO 21415-2:2015 and ISO 3093:2009, respectively.

### 2.6 Statistical analyses

The effect of the cultivar, the influence of the different environments, and their combination were evaluated by two-way analysis of variance (ANOVA) test when normality (Bera–Jarque test) criteria were satisfied. Tukey test for multiple comparison was used when significant differences (*p* < 0.05) were found in the ANOVA. Pearson correlation tests were performed between the different parameters. Several principal component analyses (PCA) were conducted on the quality traits for each wheat cultivar and each environmental condition with the aim of determining the most explanatory variables in the method, as well as environments and environmental conditions. All these analyses were performed with the XLStat (26) “add-on” for Microsoft Excel.

## 3 Results

### 3.1 Environments

The 18 environments analyzed with their particular rainfall and temperature regime (Table 2) could be grouped into three major groups based mainly in rainfall pattern: common-weather environments, dry environments, and rainy environments. However, extreme temperature events were taken into account when discussing the results.

Environments 2, 3, 6, 9, and 12 could be grouped together in the first group (common-weather environments) due to the predictable or stable distribution of temperatures and rainfall. In case of wheat in Mediterranean areas, it could be desirable to have more than 100 mm of rainfall in both winter and spring. It should be highlighted that, even when Environment 9 showed dry spring (<50 mm rainfall), the early sowing data reduced the effect of drought in the crop, being this environment considered as common-weathered in the list. In this group of environments, during the winter period, the minimum temperatures ranged between −3.5°C and 9.8°C, while rainfall registered ranged between 156.9 and 177.7 mm water. Regarding the spring, maximum temperatures ranged between 33.0°C and 37.2°C. Spring rainfall in any case exceeded 89 mm with a fairly regular rainfall distribution throughout the weeks in March and April (excepting the 40.1 mm rainfall in Environment 9).

Rainy and dry environments showed the following conditions: rainy environments (Environments 4, 11, 13, 15, and 16) showed rainfall above 115 mm in winter and 110 mm in spring, with minimum temperatures in winter between 0°C and − 2.7°C and maximum temperatures in spring below 35°C in any case. Dry environments (Environments 1, 5, 8, 10, 14, 17, and 18) were considered as such because of the low rainfall during winter and spring, which was recorded below 85 and 130 mm, respectively, in any case, with the exception of Environment 8, which had 172 mm rainfall in spring; however, due to the early sowing date in that environment, dry winter had a greater influence than common spring, due to the early development of the crop in the season. Regarding the temperatures, minimum temperatures were considered in these environments as common for the winter, but maximum spring temperatures registered values above 35°C in any case, reaching more than 38°C in two out of the six dry environments.

Special mention deserves both Environment 7 and Environment 18. Environment 7 showed a very rainy winter, with close to 240 mm rainfall, a quite dry spring (about 70 mm), and warm temperatures in winter (most days above 10°C). Regarding Environment 18, already included in the dry environment group, rainy winter (>320 mm) and dry spring (<50 mm) were registered, with warm temperatures in spring overcoming 30°C in many occasions from mid-April.

### 3.2 Effects of environment and genotype in wheat yield and quality traits of the wheat flour

The analysis of variance (ANOVA) revealed that the interaction between Cultivar and Environment had significant (*p* < 0.001) effects on wheat flour quality test (protein of the flour, W, wet gluten, P/L, and falling number—the only one with *p* < 0.01) as well as in yield parameters (yield, TKW, test weight) (Table 3). In the same way, both Cultivar and Environment significantly affected (p < 0.001) all the studied parameters. Only falling number was poorly affected by the Cultivar (*p* < 0.05). Data about grain crude protein, P and L, are presented in Supplementary Tables S1, S4 due to the high correlation of them with other studied parameters (crude protein and protein of the flour, and P and L with P/L ratio).

**Table 3:** Comparison between treatments, cultivars, and environments.

Source	W (×10^−4^ J)		Wet gluten (%)		P/L		Falling number(s)	
	DF	SS	DF	SS	DF	SS	DF	SS
Cultivar (C)	3	261973.17***	3	505.45***	3	3.82**	3	12816.79*
Environment (E)	17	722468.02***	17	1301.19***	17	2.52***	17	154418.29***
C × E	51	232266.85***	51	347.17***	51	2.52***	51	128808.92**

Source	Yield (kg ha^−1^)		TKW (g)		Test weight (kg hl^−1^)		Protein of the flour (%)	
	DF	SS	DF	SS	DF	SS	DF	SS
Cultivar (C)	3	18889245.64***	3	623.73***	3	417.46***	3	61.53***
Environment (E)	17	264206342.52***	17	4365.68***	17	530.92***	17	244.96***
C × E	51	50495199.26***	51	888.47***	51	200.74***	51	51.36***

Sum of squares and signification level (*p ≤ 0.05, **p ≤ 0.01, ***p ≤ 0.001) of the two-way ANOVA, including in the model “cultivar” and “environment” and their interaction on each parameter evaluated: yield, TKW, test weight, protein of the flour, W, wet gluten, P/L, and falling number. DF, degrees of freedom; SS, sum of squares; TKW, thousand kernel weight.

In general, as shown in Tables 4, 5, Paiva and Antequera cultivars showed the highest yield and TKW (with 6411.71 and 6172.58 kg ha^−1^, and 40.35 and 41.18 g, respectively), while Valbona and Antequera presented the best values in quality traits such as W (never lower than 255 × 10^4^ J) and P/L (0.91 and 0.73, respectively, as an average), as well as in P with more than 88 mm in both cases (Tables 6, 7 and Supplementary Table S2). The highest values in wet gluten content (29.85–47.00%), falling number (408–618 s), and protein of the flour (12.4–17.8%) were found also in Valbona cultivar, as shown in Tables 8–10, with Roxo the cultivar presenting the highest value of test weight and L, with values ranging between 79.88 and 83.59 kg hl^−1^ and 107 and 158 mm, respectively (Table 11 and Supplementary Table S3). However, this cultivar, Roxo, presented the lowest values in yield and TKW, as well as in most of the quality traits.

**Table 4:** Yield (kg ha^−1^) results affected by the interaction Cultivar × Environment.

	Yield (kg ha^−1^)				
	Antequera	Paiva	Roxo	Valbona	Average
Common					
Env. 2	8395.00 ± 623.678 a–c	7020.00 ± 704.28 b–n	6197.00 ± 1906.36 c–r	8688.50 ± 256.68 a–b	7575.10 ± 511.57 AB
Env. 3	8168.00 ± 339.41 a–e	7422.00 ± 1764.94 a–l	6884.50 ± 245.37 b–p	6491.00 ± 0.01 b–q	7241.38 ± 362.92 A–C
Env. 6	6732.00 ± 28.28 b–p	6636.50 ± 120.92 b–p	5481.00 ± 115.97 h–t	6538.50 ± 92.63 b–q	6347.00 ± 205.77 C–F
Env. 9	5665.00 ± 0.01 h–t	5741.00 ± 388.91 g–t	5044.00 ± 32.53 m–w	6208.50 ± 197.28 c–r	5664.63 ± 178.71 F–I
Env. 12	6482.49 ± 244.97 b–p	6961.72 ± 739.65 b–o	6027.49 ± 255.10 d–s	7492.00 ± 0.01 a–k	6740.92 ± 249.47 B–E
Rainy					
Env. 4	6423.50 ± 1489.87 c–q	7073.50 ± 511.24 a–m	6725.50 ± 242.54 b–p	6054.00 ± 28.28 d–s	6569.13 ± 273.73 C–F
Env. 11	8083.50 ± 176.87 a–f	8270.68 ± 346.53 a–d	7570.99 ± 283.62 a–i	8696.97 ± 503.97 a–b	8155.53 ± 191.26 A
Env. 13	7697.70 ± 60.66 a–h	7932.056 ± 30.28 a–g	7546.33 ± 813.23 a–j	7165.75 ± 288.80 a–m	7585.46 ± 167.22 AB
Env. 15	6070.50 ± 969.44 d–s	6322.50 ± 245.37 c–q	5635.50 ± 7.78 h–t	5257.00 ± 398.81 k–t	5821.38 ± 225.50 E–H
Env. 16	5050.89 ± 123.57 m–w	5371.35 ± 97.67 i–t	5303.10 ± 6.20 j–t	5174.31 ± 97.14 l–u	5226.01 ± 56.26 HI
Dry					
Env.1	5144.36 ± 370.02 m–u	6224.49 ± 248.77 c–q	4725.45 ± 533.66 o–x	5438.77 ± 694.23 i–t	5383.30 ± 262.12 G–I
Env. 5	6496.50 ± 361.33 b–q	6344.50 ± 193.04 c–q	5749.50 ± 9.19 g–t	6295.50 ± 480.13 c–q	6221.5 ± 145.44 D–G
Env. 8	6721.50 ± 85.56 b–p	8169.00 ± 455.38 a–e	6641.50 ± 639.93 b–p	5838.50 ± 119.50 f–t	6842.63 ± 358.11 B–D
Env. 10	4372.32 ± 552.75 q–y	6003.08 ± 271.36 e–s	4683.84 ± 1.47 p–x	4009.96 ± 415.25 r–y	4767.30 ± 321.95 I
Env. 14	4772.16 ± 12.07 n–x	5882.49 ± 986.13 f–s	5093.21 ± 382.24 m–v	5513.43 ± 3298.90 h–t	5315.32 ± 231.17 G–I
Env. 17	2869.00 ± 114.55 v–z	3580.00 ± 147.08 t–z	1538.00 ± 56.57 z	3825.50 ± 480.13 s–y	2953.13 ± 366.89 J
Special					
Env. 18	2657.00 ± 0.01 x–z	2945.50 ± 417.90 u–z	2190.00 ± 674.58 y–z	2834.50 ± 30.41 w–z	2656.75 ± 162.65 J
Env. 7	9305.00 ± 502.05 a	7510.50 ± 258.09 a–k	7551.50 ± 50.20 a–j	7032.50 ± 139.30 b–m	7849.88 ± 359.14 A
Average	6172.60 ± 310.25 AB	6411.70 ± 247.44 A	5588.20 ± 283.94 C	6030.80 ± 259.52B	6050.83 ± 137.11

Averages in the same row or the same column, with different lowercase letters, mean significant effect of Cultivar or Environment (*p* ≤ 0.05), respectively, according to LSD test. Uppercase letters mean significant effect of Cultivar or Environment (*p* ≤ 0.05), respectively, according to LSD test for main factors.

**Table 5:** Thousand kernel weight (TKW) (g) results affected by the interaction Cultivar × Environment.

	TKW (g)				
	Antequera	Paiva	Roxo	Valbona	Average
Common					
Env. 2	47.50 ± 1.56 b–c	46.14 ± 0.20 b–d	44.35 ± 0.35 d–j	45.75 ± 1.62 aa–ae	45.94 ± 0.56 B
Env. 3	45.20 ± 0.99 c–g	39.30 ± 1.41 n–r	37.00 ± 0.57 r–v	38.87 ± 0.01 ac–af	40.09 ± 1.27 EF
Env. 6	45.49 ± 0.65 c–f	45.95 ± 1.19 c–d	41.83 ± 0.33 j–n	42.09 ± 2.03 af–ah	43.84 ± 0.84 CD
Env. 9	38.87 ± 0.01 o–t	44.35 ± 0.07 d–j	40.25 ± 0.21 L–p	40.80 ± 0.28 c–e	41.07 ± 0.82 EF
Env. 12	29.28 ± 2.08 ae–ag	33.64 ± 0.82 x–aa	32.65 ± 0.73 aa–ad	38.87 ± 0.01 k–o	33.61 ± 1.43 GH
Rainy					
Env. 4	45.45 ± 2.05 c–f	46.90 ± 1.41 b–d	43.10 ± 0.57 f–k	36.10 ± 0.71 ad–af	42.88 ± 1.72 D
Env. 11	46.05 ± 0.21 cd	46.38 ± 1.03 b–d	44.63 ± 1.10 d–i	42.48 ± 1.45 i–m	44.88 ± 0.69 BC
Env. 13	35.40 ± 1.97 u–y	35.40 ± 1.56 u–y	35.45 ± 1.34 u–y	32.50 ± 3.96 o–t	34.69 ± 0.86 G
Env. 15	42.70 ± 0.14 g–l	43.25 ± 1.49 e–k	39.05 ± 1.77 o–s	36.20 ± 2.97 p–u	40.30 ± 1.28 EF
Env. 16	33.30 ± 0.84 y–ab	36.38 ± 0.66 t–w	30.76 ± 0.34 ab–af	30.62 ± 0.63 q–v	32.76 ± 0.98 H
Dry					
Env.1	40.98 ± 1.64 k–o	44.61 ± 0.25 d–i	36.47 ± 1.47 s–v	37.15 ± 2.48 aa–ad	39.80 ± 1.40 F
Env. 5	42.85 ± 2.19 g–k	44.75 ± 1.06 d–h	39.20 ± 0.28 o–r	37.90 ± 0.14 ae–ag	41.18 ± 1.16 E
Env. 8	42.14 ± 0.19 i–m	46.69 ± 0.27 b–d	39.66 ± 0.22 m–q	31.68 ± 0.24 b–c	40.04 ± 2.20 EF
Env. 10	32.80 ± 0.01 z–ac	42.70 ± 0.01 g–l	33.80 ± 0.01 w–aa	29.50 ± 0.01 h–l	34.70 ± 1.97 G
Env. 14	35.70 ± 0.57 u–y	35.25 ± 2.33 v–z	36.15 ± 4.60 u–x	28.50 ± 2.26 o–t	33.90 ± 1.50 GH
Env. 17	30.75 ± 0.35 ab–af	30.50 ± 0.42 ac–af	30.90 ± 0.14 ab–af	30.20 ± 0.14 u–x	30.59 ± 0.13 I
Special					
Env. 18	38.87 ± 0.01 o–t	27.30 ± 0.14 ag–ah	26.55 ± 0.50 ah–ai	24.00 ± 0.14 u–x	29.18 ± 2.32 J
Env. 7	52.88 ± 0.36 a	51.83 ± 0.59 a	48.67 ± 0.19 b	47.78 ± 0.28 ai	50.29 ± 0.86 A
Average	40.35 ± 1.08 B	41.18 ± 1.11 A	37.80 ± 0.95 C	36.17 ± 1.08 D	38.88 ± 0.54

Averages in the same row or the same column, with different lowercase letters, mean significant effect of Variety or Environment (*p* ≤ 0.05), respectively, according to LSD test. Uppercase letters mean significant effect of Cultivar or Environment (*p* ≤ 0.05), respectively, according to LSD test for main factors.

**Table 6:** Deformation work or W (×10^−4^ J) results affected by the interaction Cultivar × Environment.

	W (×10^−4^ J)				
	Antequera	Paiva	Roxo	Valbona	Average
Common					
Env. 2	297.20 ± 3.96 n–u	167.00 ± 9.90 af	224.35 ± 27.79 w–ad	259.35 ± 20.72 s–z	236.97 ± 20.04 G
Env. 3	271.95 ± 5.73 r–x	224.85 ± 6.86 v–ad	175.90 ± 2.69 ad–af	310.67 ± 0.01 j–s	245.84 ± 20.46 G
Env. 6	227.65 ± 16.05 v–ad	168.50 ± 9.19 ae–af	179.15 ± 13.93 ad–af	220.50 ± 45.96 x–ae	198.95 ± 12.67 H
Env. 9	310.67 ± 0.01 j–s	247.00 ± 63.64 u–ab	308.00 ± 22.63 k–t	292.50 ± 53.03 o–u	289.54 ± 16.03 E
Env. 12	419.30 ± 27.29 d–e	362.20 ± 18.67 f–j	297.65 ± 7.99 m–u	310.67 ± 0.01 j–s	347.45 ± 19.99 D
Rainy					
Env. 4	277.50 ± 0.71 p–v	202.75 ± 8.13 aa–af	303.00 ± 5.66 l–t	345.50 ± 4.95 i–n	282.19 ± 21.03 EF
Env. 11	273.45 ± 0.78 q–w	305.95 ± 23.41 l–t	195.30 ± 1.84 ab–af	333.55 ± 4.88 i–o	277.06 ± 20.20 EF
Env. 13	417.50 ± 23.33 d–e	325.50 ± 10.61 i–q	302.50 ± 40.31 l–t	399.00 ± 32.53 e–h	361.13 ± 21.18 CD
Env. 15	262.70 ± 7.50 s–y	233.50 ± 17.68 v–ac	199.00 ± 14.14 ab–af	297.00 ± 9.90 n–u	248.05 ± 15.07 G
Env. 16	428.00 ± 16.97 c–e	360.00 ± 15.56 f–k	352.75 ± 27.93 g–l	513.50 ± 71.42 a–b	413.56 ± 28.55 B
Dry					
Env.1	304.10 ± 0.85 L–t	165.50 ± 1.13 af	181.40 ± 20.22 ac–af	339.10 ± 2.69 i–o	247.53 ± 30.66 G
Env. 5	271.00 ± 14.14 r–x	215.00 ± 4.24 y–af	188.50 ± 20.51 ac–af	291.50 ± 0.71 o–u	241.50 ± 17.14 G
Env. 8	411.00 ± 7.07 d–f	315.50 ± 30.41 j–r	295.00 ± 28.28 n–u	426.00 ± 36.77 c–e	361.88 ± 24.50 CD
Env. 10	375.75 ± 2.33 e–i	205.40 ± 9.90 aa–af	320.90 ± 27.58 j–r	517.10 ± 20.79 a–b	354.79 ± 45.58 CD
Env. 14	527.50 ± 10.60 a	350.00 ± 50.91 h–m	329.00 ± 28.28 i–p	561.50 ± 41.72 a	442.00 ± 43.07 A
Env. 17	474.00 ± 9.90 b–c	339.50 ± 17.68 i–o	376.50 ± 4.95 e–i	456.25 ± 31.46 c–d	411.60 ± 23.06 B
Special					
Env. 18	310.67 ± 0.01 j–s	404.25 ± 49.85 d–g	363.00 ± 38.18 f–j	419.25 ± 72.48 d–e	374.29 ± 21.85 C
Env. 7	270.80 ± 2.55 r–x	208.00 ± 42.43 z–af	297.35 ± 44.76 m–u	255.35 ± 11.81 t–aa	257.88 ± 15.90 FG
Average	340.60 ± 14.38 B	266.69 ± 13.43 C	271.63 ± 12.01 C	363.79 ± 17.05 A	310.68 ± 7.87

Averages in the same row or the same column, with different lowercase letters, mean significant effect of Variety or Environment (*p* ≤ 0.05), respectively, according to LSD test. Uppercase letters mean significant effect of Cultivar or Environment (*p* ≤ 0.05), respectively, according to LSD test for main factors.

**Table 7:** Tenacity by extensibility ratio or P/L results affected by the interaction Cultivar × Environment.

	P/L				
	Antequera	Paiva	Roxo	Valbona	Average
Common					
Env. 2	0.90 ± 0.04 d–h	0.69 ± 0.13 i–o	0.70 ± 0.10 h–n	1.41 ± 0.17 b	0.92 ± 0.12 A
Env. 3	0.68 ± 0.06 i–p	0.66 ± 0.06 i–p	0.39 ± 0.03 s–u	0.67 ± 0.01 i–p	0.60 ± 0.05 D–F
Env. 6	0.94 ± 0.14 c–g	0.51 ± 0.16 n–u	0.52 ± 0.03 n–u	1.71 ± 0.09 a	0.92 ± 0.20 A
Env. 9	0.67 ± 0.01 i–p	0.55 ± 0.12 m–t	0.40 ± 0.06 r–u	0.75 ± 0.11 g–m	0.59 ± 0.06 EF
Env. 12	0.74 ± 0.05 h–m	0.73 ± 0.01 h–m	0.67 ± 0.05 i–p	0.67 ± 0.01 i–p	0.70 ± 0.02 BC
Rainy					
Env. 4	0.99 ± 0.26 c–f	0.62 ± 0.02 l–q	0.62 ± 0.02 l–q	0.71 ± 0.05 h–n	0.73 ± 0.07 B
Env. 11	0.66 ± 0.13 i–p	0.84 ± 0.11 d–i	0.51 ± 0.13 n–u	0.90 ± 0.11 d–h	0.72 ± 0.07 B
Env. 13	0.60 ± 0.03 m–r	0.43 ± 0.07 q–u	0.36 ± 0.02 t–u	0.51 ± 0.03 n–u	0.47 ± 0.04 G
Env. 15	0.84 ± 0.04 d–i	0.51 ± 0.01 n–u	0.50 ± 0.11 o–u	1.03 ± 0.20 c–d	0.72 ± 0.10 B
Env. 16	0.63 ± 0.01 k–q	0.56 ± 0.06 m–t	0.56 ± 0.06 m–s	0.98 ± 0.09 c–f	0.68 ± 0.07 B–E
Dry					
Env.1	0.53 ± 0.09 n–u	0.56 ± 0.05 m–t	0.38 ± 0.01 s–u	0.98 ± 0.24 c–f	0.61 ± 0.10 C–F
Env. 4	0.99 ± 0.26 c–f	0.62 ± 0.02 l–q	0.62 ± 0.02 l–q	0.71 ± 0.05 h–n	0.73 ± 0.07 B
Env. 5	0.63 ± 0.06 j–q	0.52 ± 0.04 n–u	0.36 ± 0.01 t–u	0.82 ± 0.13 f–l	0.58 ± 0.07 E–G
Env. 8	0.73 ± 0.04 h–m	0.71 ± 0.06 h–n	0.37 ± 0.03 s–u	0.98 ± 0.13 c–f	0.70 ± 0.09 B–D
Env. 10	0.49 ± 0.16 o–u	0.37 ± 0.15 s–u	0.387 ± 0.12 s–u	0.82 ± 0.27 e–k	0.51 ± 0.09 FG
Env. 14	0.56 ± 0.18 m–s	0.44 ± 0.06 q–u	0.34 ± 0.01 u	0.83 ± 0.09 d–j	0.54 ± 0.08 FG
Env. 17	0.82 ± 0.08 f–l	0.56 ± 0.01 m–t	0.44 ± 0.01 q–u	1.02 ± 0.07 c–e	0.71 ± 0.09 BC
Special					
Env. 18	0.67 ± 0.01 i–p	0.48 ± 0.06 p–u	0.40 ± 0.04 r–u	0.55 ± 0.08 m–t	0.53 ± 0.04 FG
Env. 7	0.99 ± 0.09 c–f	0.55 ± 0.06 m–t	0.90 ± 0.12 d–h	1.14 ± 0.17 c	0.90 ± 0.09 A
Average	0.73 ± 0.03 B	0.57 ± 0.0.02 C	0.49 ± 0.03 D	0.91 ± 0.05 A	0.68 ± 0.02

Averages in the same row or the same column, with different lowercase letters, mean significant effect of Variety or Environment (*p* ≤ 0.05), respectively, according to LSD test. Uppercase letters mean significant effect of Cultivar or Environment (*p* ≤ 0.05), respectively, according to LSD test for main factors.

**Table 8:** Wet gluten content (%) results affected by the interaction Cultivar × Environment.

	Wet gluten (%)				
	Antequera	Paiva	Roxo	Valbona	Average
Common					
Env. 2	28.95 ± 0.35 aa–ae	26.75 ± 0.49 ae_af	30.75 ± 0.07 u–ad	29.85 ± 2.33 x–ad	29.08 ± 0.69 K
Env. 3	30.25 ± 0.35 v–ad	31.35 ± 1.21 s–ab	33.30 ± 0.99 m–u	33.71 ± 0.01 l–t	320.15 ± 0.62 H
Env. 6	29.65 ± 2.33 z–ad	29.65 ± 0.49 z–	30.70 ± 0.57 u–ad	32.60 ± 1.41 o–w	30.65 ± 0.63 IJ
Env. 9	33.71 ± 0.01 L–t	30.15 ± 1.34 w–ad	31.25 ± 2.76 s–ab	32.40 ± 0.57 p–y	31.88 ± 0.70 HI
Env. 12	33.85 ± 1.77 k–t	32.30 ± 0.01 q–z	38.15 ± 3.18 cdefg	33.71 ± 0.01 l–t	34.50 ± 1.03 EF
Rainy					
Env. 4	26.02 ± 0.53 af	28.90 ± 1.13 aa–ae	28.87 ± 0.37 ab–ae	35.03 ± 1.23 h–p	29.71 ± 1.35 JK
Env. 11	31.20 ± 2.69 t–ac	32.39 ± 0.43 p–y	35.85 ± 1.91 g–m	37.05 ± 1.48 e–i	34.12 ± 1.10 E–G
Env. 13	36.75 ± 1.48 f–j	29.80 ± 1.56 x–ad	32.70 ± 1.41 o–w	40.90 ± 0.99 b	35.04 ± 1.74 DE
Env. 15	31.60 ± 0.42 r–aa	31.40 ± 0.42 s–ab	32.50 ± 0.99 o–x	37.15 ± 0.21 e–i	33.16 ± 0.96 F–H
Env. 16	36.90 ± 0.14 e–j	32.63 ± 2.30 o–w	36.05 ± 0.78 g–l	40.43 ± 1.03 bc	36.50 ± 1.98 C
Dry					
Env.1	34.30 ± 0.85 j–r	28.50 ± 1.41 ac–af	34.55 ± 1.34 i–q	34.55 ± 2.19 i–q	32.98 ± 1.13 GH
Env. 5	32.45 ± 1.91 o–y	30.70 ± 0.99 u–ad	33.95 ± 0.78 k–s	37.05 ± 0.49 e–i	33.54 ± 1.00 FG
Env. 8	35.12 ± 0.31 h–o	33.13 ± 0.31 m–u	35.07 ± 1.01 h–p	37.31 ± 0.28 e–h	35.16 ± 0.62 C–E
Env. 10	33.30 ± 0.99 m–u	29.75 ± 1.34 yz–ad	34.93 ± 1.80 h–q	34.28 ± 3.92 j–r	33.06 ± 1.04 GH
Env. 14	40.75 ± 0.07 bc	36.55 ± 2.33 f–k	41.60 ± 0.14 b	47.00 ± 0.71 a	41.48 ± 1.54 A
Env. 17	39.55 ± 0.06 b–e	35.61 ± 1.15 g–n	39.04 ± 0.23 b–f	37.62 ± 1.73 d–h	37.95 ± 0.68 B
Special					
Env. 18	33.71 ± 0.01 l–t	32.95 ± 2.76 n–v	37.50 ± 0.01 e–h	40.30 ± 0.28 b–d	36.12 ± 1.26 CD
Env. 7	29.20 ± 0.14 aa–ae	28.40 ± 1.56 ad–af	30.20 ± 1.56 w–ad	31.25 ± 1.34 s–ab	29.76 ± 0.57 JK
Average	33.18 ± 0.65 C	31.16 ± 0.45 B	34.28 ± 0.59 B	36.23 ± 0.71 A	33.71 ± 0.34

Averages in the same row or the same column, with different lowercase letters, mean significant effect of Variety or Environment (*p* ≤ 0.05), respectively, according to LSD test. Uppercase letters mean significant effect of Cultivar or Environment (*p* ≤ 0.05), respectively, according to LSD test for main factors.

**Table 9:** Falling number results affected by the interaction Cultivar × Environment.

	Falling number				
	Antequera	Paiva	Roxo	Valbona	Average
Common					
Env. 2	364.00 ± 127.28 l–n	405.00 ± 26.87 i–n	463.50 ± 2.12 c–i	455.50 ± 10.61 d–k	442.00 ± 24.75 F–H
Env. 3	465.00 ± 12.73 c–i	428.00 ± 42.43 d–m	462.50 ± 6.36 c–i	453.88 ± 0.01 d–k	452.35 ± 8.71 D–F
Env. 6	429.00 ± 49.50 d–m	442.00 ± 31.11 d–k	435.50 ± 40.31 d–k	441.00 ± 29.70 d–k	436.88 ± 11.19 E–G
Env. 9	453.88 ± 0.01 c–j	387.00 ± 52.33 j–n	378.50 ± 4.95 k–n	413.50 ± 38.89 d–k	408.22 ± 15.10 GH
Env. 12	518.00 ± 46.67 b–c	471.00 ± 55.15 c–i	462.50 ± 75.66 c–i	453.88 ± 0.01 d–k	476.35 ± 17.99 B–D
Rainy					
Env. 4	340.00 ± 19.80 n	358.50 ± 24.75 mn	402.25 ± 33.59 i–n	467.50 ± 0.71 d–k	392.06 ± 20.89 H
Env. 11	434.50 ± 14.85 d–l	450.50 ± 10.61 c–j	440.50 ± 12.02 d–k	458.50 ± 13.44 d–k	446.00 ± 5.22 D–F
Env. 13	484.35 ± 24.54 b–g	520.50 ± 26.16 b–c	486.50 ± 3.54 b–f	467.00 ± 2.83 d–k	489.59 ± 9.38 BC
Env. 15	438.50 ± 28.99 d–k	496.75 ± 30.05 b–d	435.75 ± 25.81 d–k	461.00 ± 43.84 d–k	458.00 ± 13.63 C–E
Env. 16	465.50 ± 36.06 c–i	470.25 ± 27.22 c–i	467.50 ± 33.23 c–i	460.00 ± 19.80 d–k	465.81 ± 8.63 C–E
Dry					
Env.1	491.00 ± 49.50 b–e	462.00 ± 2.83 c–i	429.00 ± 25.46 d–m	439.50 ± 0.71 d–k	455.38 ± 12.48 C–F
Env. 5	497.50 ± 24.75 b–d	424.50 ± 44.55 e–m	425.00 ± 21.21 e–m	460.00 ± 25.46 d–k	451.75 ± 14.94 D–F
Env. 8	425.00 ± 26.87 e–m	466.00 ± 5.56 c–i	430.50 ± 13.44 d–l	456.00 ± 2.83 d–k	444.68 ± 8.18 D–F
Env. 10	614.00 ± 38.18 a	424.00 ± 33.94 e–m	498.50 ± 24.75 b–d	614.00 ± 4.24 d–k	537.63 ± 33.64 A
Env. 14	548.00 ± 35.36 a–b	471.00 ± 93.34 c–i	476.50 ± 64.35 c–h	513.00 ± 29.70 d–k	502.17 ± 21.51 B
Env. 17	492.00 ± 29.70 b–e	493.50 ± 20.51 b–e	417.50 ± 37.48 f–m	430.25 ± 31.47 d–k	458.31 ± 16.51 C–E
Special					
Env. 18	453.88 ± 0.01 c–j	424.50 ± 45.96 e–m	414.00 ± 25.46 g–m	436.00 ± 29.70 d–k	432.10 ± 10.49 E–G
Env. 7	453.00 ± 7.07 c–j	469.00 ± 53.74 c–i	433.50 ± 44.55 d–l	408.50 ± 9.19 d–k	441.00 ± 13.62 EG
Average	464.80 ± 11.53 A	448.00 ± 8.35 BC	442.19 ± 6.56 C	460.50 ± 7.88 AB	453.87 ± 4.36

Averages in the same row or the same column, with different lowercase letters, mean significant effect of Variety or Environment (*p* ≤ 0.05), respectively, according to LSD test. Uppercase letters mean significant effect of Cultivar or Environment (*p* ≤ 0.05), respectively, according to LSD test for main factors.

**Table 10:** Protein of the flour (%) results affected by the interaction Cultivar × Environment.

	Protein (in flour) (g)				
	Antequera	Paiva	Roxo	Valbona	Average
Common					
Env. 2	12.20 ± 0.07 v–y	10.95 ± 0.35 z	13.45 ± 0.92 n–u	13.20 ± 0.57 o–v	12.45 ± 0.44 H
Env. 3	12.70 ± 0.37 t–x	12.25 ± 0.35 v–y	13.10 ± 0.14 p–v	14.09 ± 0.01 j–q	13.03 ± 0.29 G
Env. 6	11.90 ± 0.84 w–z	12.20 ± 0.28 v–y	12.25 ± 0.64 v–y	12.40 ± 0.01 u–x	12.19 ± 0.17 H
Env. 9	14.09 ± 0.01 j–q	12.54 ± 0.50 t–x	13.57 ± 0.62 m–t	13.03 ± 0.67 q–v	13.31 ± 0.27 FG
Env. 12	14.87 ± 0.10 h–l	14.60 ± 0.35 h–m	15.09 ± 0.41 g–k	14.09 ± 0.01 j–q	14.66 ± 0.18 DE
Rainy					
Env. 4	11.15 ± 1.06 y–z	11.63 ± 0.11 x–z	12.78 ± 0.18 t–w	14.15 ± 0.78 j–p	12.43 ± 0.51 H
Env. 11	14.05 ± 1.49 k–r	13.65 ± 0.35 m–t	14.40 ± 0.28 i–n	15.65 ± 0.92 d–h	14.44 ± 0.40 E
Env. 13	14.60 ± 0.85 h–m	13.20 ± 0.11 o–v	14.05 ± 0.35 k–r	16.35 ± 0.35 c–e	14.55 ± 0.49 E
Env. 15	12.90 ± 0.72 s–w	12.95 ± 0.28 r–w	13.05 ± 0.92 p–v	14.45 ± 0.21 i–n	13.39 ± 0.32 FG
Env. 16	14.99 ± 0.71 g–l	14.79 ± 0.11 h–l	14.96 ± 0.06 g–l	16.58 ± 0.11 c–d	15.33 ± 0.30 C
Dry					
Env.1	14.25 ± 0.07 j–o	12.10 ± 0.28 v–y	14.50 ± 0.57 i–n	13.90 ± 1.13 l–s	13.69 ± 0.42 F
Env. 5	13.05 ± 0.67 p–v	12.85 ± 0.50 s–w	13.45 ± 0.78 n–u	14.90 ± 0.28 h–l	13.56 ± 0.36 FG
Env. 8	15.14 ± 0.51 g–k	14.48 ± 0.74 i–n	14.66 ± 0.35 h–m	15.49 ± 0.12 d–i	14.94 ± 0.21 C–E
Env. 10	15.20 ± 0.14 f–j	13.05 ± 0.28 p–v	15.20 ± 0.28 f–j	17.85 ± 0.92 a–b	15.33 ± 0.70 C
Env. 14	16.30 ± 0.42 c–f	15.40 ± 0.50 e–i	16.30 ± 0.57 c–f	18.90 ± 0.42 a	16.73 ± 0.55 A
Env. 17	16.87 ± 0.16 b–c	15.16 ± 0.74 g–k	16.44 ± 0.37 c–e	16.03 ± 0.18 c–g	16.12 ± 0.28 B
Special					
Env. 18	14.09 ± 0.01 j–q	14.42 ± 0.37 i–n	15.51 ± 1.26 d–i	16.46 ± 0.92 c–e	15.12 ± 0.46 CD
Env. 7	12.32 ± 0.02 v–x	12.12 ± 0.11 v–y	12.37 ± 0.24 u–x	13.02 ± 0.03 q–w	12.46 ± 0.14 H
Average	13.93 ± 0.27 B	13.24 ± 0.22 C	14.17 ± 0.22 B	15.03 ± 0.30 A	14.09 ± 0.14

Averages in the same row or the same column, with different lowercase letters, mean significant effect of Variety or Environment (*p* ≤ 0.05), respectively, according to LSD test. Uppercase letters mean significant effect of Cultivar or Environment (*p* ≤ 0.05), respectively, according to LSD test for main factors.

**Table 11:** Test weight (g) results affected by the interaction Cultivar × Environment.

	Test weight (g)				
	Antequera	Paiva	Roxo	Valbona	Average
Common					
Env. 2	82.44 ± 0.80 h–o	81.39 ± 0.87 l–w	80.16 ± 0.23 u–ac	81.06 ± 0.08 o–y	81.26 ± 0.37 CD
Env. 3	84.85 ± 0.21 a–c	85.35 ± 0.50 a	84.60 ± 0.14 a–d	80.94 ± 0.01 o–z	83.94 ± 0.71 A
Env. 6	82.14 ± 0.20 i–q	82.80 ± 0.85 e–m	82.48 ± 0.67 g–o	80.92 ± 0.59 o–z	82.08 ± 0.34 B
Env. 9	80.94 ± 0.01 o–z	82.75 ± 0.59 f–n	83.59 ± 0.18 b–i	81.11 ± 0.68 n–y	82.10 ± 0.47 B
Env. 12	80.60 ± 0.14 q–aa	79.55 ± 1.34 y–ac	81.35 ± 0.92 l–w	80.94 ± 0.01o–z	80.61 ± 0.36 DE
Rainy					
Env. 4	79.68 ± 1.67 x–ac	81.55 ± 0.16 l–v	81.75 ± 0.31 k–u	76.45 ± 0.63 af	79.86 ± 0.90 EF
Env. 11	83.85 ± 0.22 a–h	84.42 ± 0.83 a–e	85.11 ± 0.56 a–b	81.87 ± 0.89 j–s	83.81 ± 0.52 A
Env. 13	79.97 ± 0.05 v–ac	80.20 ± 0.28 t–ac	82.05 ± 0.06 i–q	74.20 ± 0.28 ag	79.10 ± 1.19 FG
Env. 15	82.18 ± 0.25 i–q	81.91 ± 0.57 j–s	83.63 ± 0.61 b–i	78.85 ± 0.49 ab–ad	81.64 ± 0.72 BC
Env. 16	79.33 ± 0.32 z–ad	77.72 ± 0.10 ad–af	79.88 ± 0.39 w–ac	72.30 ± 1.41 ah	77.30 ± 1.23 H
Dry					
Env.1	82.30 ± 1.84 h–p	83.50 ± 0.71 b–j	82.00 ± 1.41 i–r	80.12 ± 1.25 u–ac	81.98 ± 0.65 BC
Env. 5	83.35 ± 0.49 c–k	82.16 ± 0.23 i–q	83.33 ± 0.46 c–k	79.04 ± 0.23 aa–ad	81.97 ± 0.72 BC
Env. 8	81.82 ± 0.17 k–t	83.00 ± 0.57 d–l	83.59 ± 0.40 b–i	77.11 ± 1.13 ae_af	81.38 ± 1.05 B–D
Env. 10	79.06 ± 0.08 aa–ad	80.85 ± 1.20 o–z	80.72 ± 1.02 p–z	71.39 ± 0.16 ah	78.00 ± 1.59 H
Env. 14	81.61 ± 0.16 L–v	80.32 ± 2.29 s–ab	81.43 ± 1.32 L–w	74.78 ± 0.31 ag	79.54 ± 1.19 FG
Env. 17	79.32 ± 0.59 z–ad	80.36 ± 0.79 r–ab	80.96 ± 0.78 o–z	76.64 ± 0.76 af	79.32 ± 0.70 FG
Special					
Env. 18	80.94 ± 0.01 o–z	78.58 ± 2.68 ac–ae	81.33 ± 0.50 m–x	74.80 ± 2.10 ag	78.91 ± 1.16 G
Env. 7	84.09 ± 0.41 a–g	84.30 ± 0.57 a–f	84.49 ± 0.71 a–d	82.38 ± 0.37 h–p	83.81 ± 0.37 A
Average	81.58 ± 0.30 B	81.70 ± 0.37 B	82.36 ± 0.28 A	78.05 ± 0.58 C	80.92 ± 0.24

Averages in the same row or the same column, with different lowercase letters, mean significant effect of Variety or Environment (*p* ≤ 0.05), respectively, according to LSD test. Uppercase letters mean significant effect of Cultivar or Environment (*p* ≤ 0.05), respectively, according to LSD test for main factors.

Taking into account the description of the environments given above, it is interesting to highlight that Environment 7 favors better yield (not showing values below 7,000 kg ha^−1^), TKW, and test weight (Tables 4, 5, 11), while Environment 14 showed the best results for most of the quality traits (protein of the flour, W, falling number, and wet gluten) (Tables 6, 8–10, respectively). Contrary to this, Environment 18 brought the lowest yield and TKW values, not reaching 3,000 kg ha^−1^ in any of the four studied cultivars and being below 40 g of TKW (Tables 4, 5). Regarding the quality traits, Environments 2 and 6 were the two situations where most of the quality traits showed the lowest values (Tables 6–11 and Supplementary Tables S2–S4).

Analyzing the data of each cultivar regarding the Environment, Antequera cultivar showed the best yield, TKW, and test weight data in Environment 7, as well as what happened with cultivars Paiva and Roxo, presenting yield values greater than 7,500 kg ha^−1^ and more than 9,300 kg ha^−1^ in Antequera. However, the environments where Valbona cultivar showed the highest yield were Environment 2 and 11, overcoming the 8,600 kg ha^−1^ (Tables 4, 5, 11), even though there were no significant differences with Environment 7 (7,032 kg ha^−1^), where the best yield component data were recorded (>82.00 kg hl^−1^ in test weight and 47.78 g for TKW). Regarding the quality, Antequera, Paiva, and Valbona cultivars exhibited the best results for the studied quality traits in Environment 14, with, among others, protein of the flour values above 15.4% and W values above 329 × 10^4^ J, while Roxo cultivar expressed their best values of quality traits in Environments 17 and 18, presenting more than 16% of protein in the flour and above 400 × 10^4^ J of W (Tables 6, 10).

As it can be inferred from the previous paragraphs, it is usual that the environments where the cultivars promote the best yields are not those that promote the best quality of the wheat grain/flour. Thus, a negative correlation was found between the yield and the protein of the flour, as well as between the yield and the W (Table 12). As expected, test weight was highly and positively correlated with yield and TKW, so it is not surprising that a high negative correlation was found between test weight and protein of the flour (*r* = 0.60***) and W (*r* = 0.70***) on the one hand and between TKW and protein of the flour (*r* = 0.75***) and W (*r* = 0.67***) on the other hand (Table 12).

**Table 12:** Linear relationship of the wheat quality parameters in the experiments, and the quality traits and climatic conditions.

Parameter combination	Expression	*r*	*p*
Crude Protein × Protein flour	*y* = 0.9102*x* + 0.0797	0.88	***+
Protein flour × Wet gluten	*y* = 2.1272*x* + 3.7355	0.88	***+
Protein flour × TKW	*y* = −2.9937*x* + 81.061	0.75	***−
Test weight × TKW	*y* = 1.4639*x* − 79.61	0.65	***+
W × TKW	*y* = −0.0465*x* + 53.333	0.67	***−
Test weight × Protein flour	*y* = −0.3363*x* + 41.307	0.60	***−
Test weight × W	*y* = −22.467*x* + 2129.1	0.70	***−
Test weight × Yield	*y* = 234.24*x* − 12,880	0.43	**+
Protein flour × W	*y* = 45.306*x* − 327.79	0.79	***+
Protein flour × Yield	*y* = −483.75*x* + 12,896	0.50	***−
W × Yield	*y* = −6.7325*x* + 8170.3	0.40	***−
W × Winter irrigation	*y* = 0.1801*x* − 38.51	0.70	***
Protein flour × Winter irrigation	*y* = 9.6667*x* − 118.78	0.70	***
Wet gluten × Winter irrigation	*y* = 4.0884*x* − 120.38	0.68	***
Protein flour × Total rainfall	*y* = −44.697*x* + 1097.5	0.57	*
Wet gluten × Total rainfall	*y* = −19.829*x* + 1136.1	0.58	**

***means probability of <0.001.

Wet gluten and protein content of the flour, as well as W and protein of the flour, showed to be strongly and positively correlated (*r* = 0.88*** and *r* = 0.79***, respectively), aside from wet gluten and protein of the flour with the total rainfall of the year along the experiment with *r* = 0.58** and *r* = 0.57*, respectively (Table 12).

**Figure 1:**
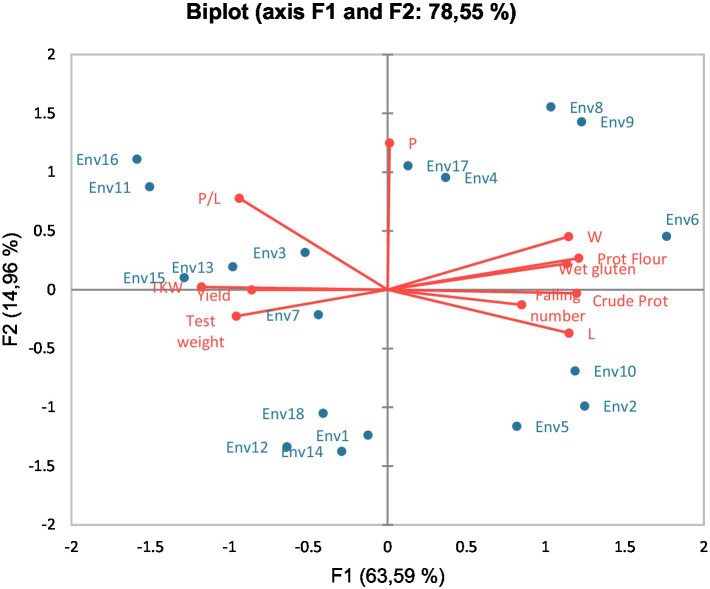
Principal component analysis (PCA) biplot of wheat quality traits and studied environments.

**Figure 2:**
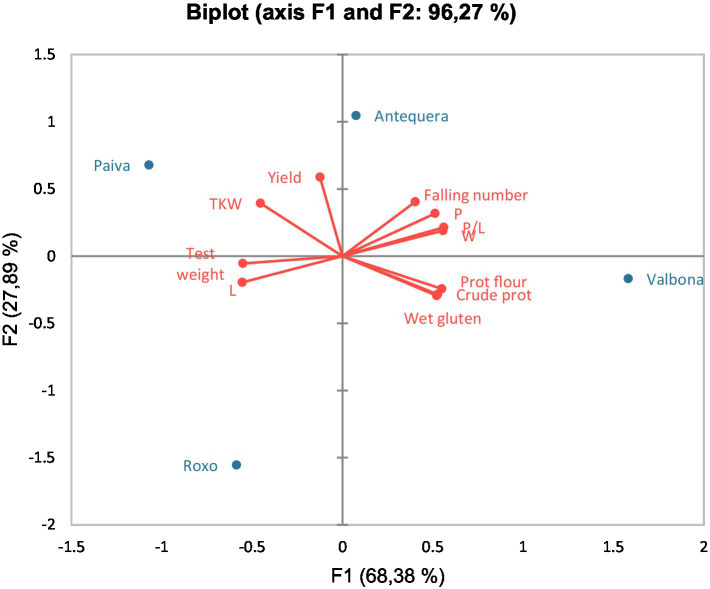
Principal component analysis (PCA) biplot of wheat quality traits and studied cultivars.

**Figure 3:**
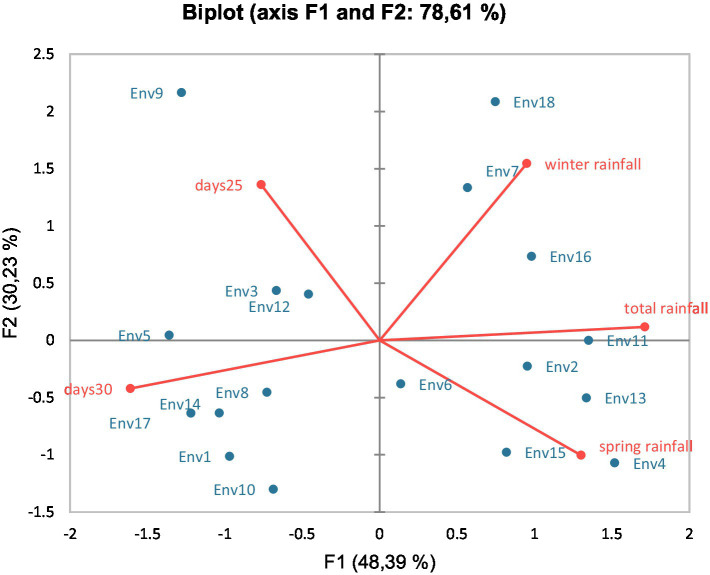
Principal component analysis (PCA) biplot of environments and some climatic characteristics.

Principal component analysis (PCA) of the data presented in Figure 1 related yield and quality traits to the Environments. Variability was explained, in this case, in more than 78.5%, being Environment 7, 13, and 15 well and positively related to yield and yield components (TKW and test weight) as explained by axis F1. In case of cultivars, variability was explained in this PCA by more than 96% (Figure 2), relating Paiva and Antequera with yield and TKW, and Valbona with some quality traits such as protein, W, or P/L. Talking about the connection between the environment and the climate data, PCA presented in Figure 3 explained close to 80% of the variability. Environments 14 and 17 related closely and positively to the number of days above 30°C in spring; Environment 7 was quite well characterized by winter rainfall; and Environments 4, 11, and 15 were previously considered as rainy environments, all of them related to total or spring rainfall.

## 4 Discussion

### 4.1 Climate traits influence on wheat yield and quality

According to recent IPCC reports (27), the warming of the climate due to climate change is unequivocal, and variables such as temperature and precipitation and their new patterns will show a great impact on agriculture, affecting global food security (28, 29). That is the reason why the responses of wheat crop (as part of the group of the most important staple crops worldwide) to climate change have gained extensive attention in recent years (30). In our experiment, four bread wheat cultivars obtained and registered in different years, representing the main lines of germplasm of the area, were tested in 18 different environments regarding temperatures and rainfall. Among these environments, almost half of them could be considered as not common for the area, showing extremely dry winter or spring, very hot springs, or registering heavy rain episodes in a short time followed by serious drought periods during critical growth phases (Supplementary Figures S1, S5, S7, S8, S10, S13–S16, S18). Taking into account the new climatic scenarios that are coming in the near future, the information given in this work could give us an idea about the behavior of some cultivars or germplasm representatives regarding their resilience to climate change. As presented in the Results section, the analysis of variance (ANOVA) revealed that the interaction between Cultivar and Environment had significant (*p* < 0.001) effects on each yield and quality trait that was studied (Table 3), which is in accordance with previous research (31).

Even though the four bread wheat cultivars tested here have been used by farmers for years and were registered after being tested in different environments, it is necessary to highlight that the rapidly changing environment type due to climate change is putting the previously tested cultivars, which were proven to be suitable for temperatures and rainfall ranges that are being recently overtaken. Regarding yield, according to Semenov and Stratonovitch’s (32) studies, the two main factors that contribute to yield increase are the improvement in light conversion efficiency and the lengthening of the grain filling period. The improvement of both factors results in a better harvest index, influenced by an optimal phenology. Thus, the quite low yield results in most of the cultivars tested in our study (Tables 4, 5) in Environments 1, 5, 14, and 18 (which were classified as dry environments, Supplementary Figures S1, S5, S14, S18) could be explained by the scarce rainfall during spring, especially in early spring (March and early April), which induced a shortening in the grain filling period, affecting both yield (Table 4) and TKW (Table 5). This fact is very important because, even though FAO (33) has registered a continuous increase in wheat yield in Europe, attributing the improvement to better agronomic practices and, especially, to wheat breeding programs and the achievements of new cultivars (34), climate change, with predicted scenarios of raising temperatures and decreasing rainfall, is threatening wheat yields. Thus, Zhang et al. (10) stated in their experiments that wheat yields could decline by 3.6% per 1°C warming. In addition to warming, heat stress with exceptional high temperatures during days or hours, as the one that occurred in the spring of Environment 18, is becoming more common to be coupled with drought (35), reducing severely grain filling capacity and so yield and TKW.

### 4.2 Breeding as a tool to maintain wheat yield and quality

Tolerance to heat stress is defined as the ability to maintain grain yield following a heat episode (36). There are three principal ways in which such tolerance can be achieved. Firstly, crops may maintain the duration of grain filling and, thus, the duration of resource capture and translocation of assimilates from leaves to grains (37). Secondly, the plants should be able to maintain the assimilation of carbohydrates and nutrients, which requires maintenance of the green leaf area (GLA) (38). Thirdly, the plants may remobilize stem water-soluble carbohydrates (WSC) from stems to supplement the lack of net new assimilation of carbohydrates from photosynthesis (39). In this sense, breeding should take this fact into account when selecting germplasm (40), preferably with a higher rate of grain filling. However, not only the cultivars can be selected or modified to adapt wheat crop to climate, but also crop management practices. Thus, Asseng et al. (41) and Moniruzzaman et al. (42) stated that changing planting dates was adopted by farmers as a favorable measurement to minimize the effects of extreme weather episodes; nevertheless, many times, the farmer should adapt the sowing date to climate and soil conditions, and sowing is taking place later than desired. In our study, sowing date took place quite early in Environments 1, 7, and 8, strongly affecting the final yield. In Environments 1 and 8, after sowing, a quite dry winter took place, reducing the tilling capacity and so, the final yield due to the importance of spike number in the final yield (43) (Supplementary Figures S1, S8 and Table 4). Conversely, early sowing in Environment 7 enabled crop wheat to present a fast and correct development due to rainy winter, facilitating the crop to escape from the effects of quite dry spring (Supplementary Figure S7). In fact, even when in this Environment only 70 mm rainfall occurred, it stood out as the best Environment regarding yield, which was expected taking into account the PCA of the Environments with the yield data (Figure 3).

Regarding the cultivars, it is interesting to highlight that Paiva and Antequera were the cultivars showing the best yield results in many of the Environments, with Roxo the cultivar registering the lowest yield values (Table 4). This could be explained by the year of registration for the cultivars; thus, while Antequera and Paiva were registered in 2009 and 2016, respectively (relatively new cultivars), Roxo has been a commercial cultivar since 1995, which makes this cultivar to be overtaken by newer cultivars. Figure 2 also supports this fact because, while Paiva and Antequera are related to yield and TKW in the PCA developed for cultivars and yield/quality traits, Roxo appears to be far more related to any interesting trait by farmers or industry. In literature, Roxo and Paiva have been previously compared, and Roxo was found to show no yield differences with Paiva; however, Luís et al. (44) carried out their study in only one season. This fact supports the idea of the necessity of having a considerable batch of years to really test the cultivar behavior over time. Antequera cultivar was expected to be found among the best yield cultivars due to being considered an “improver” cultivar, which, according to Palminha (45), means a cultivar that over the years has been able to produce better grain yield and quality (especially protein content) than the most common cultivars sown in the area.

This makes us link with the quality traits; thus, despite today’s importance of food quality, wheat grain quality has historically received much less attention than yield, both in breeding programs and in literature, even though it is a critical aspect of human nutrition. Thereby, grain protein content is not only a quality trait affecting directly wheat nutritional quality, but also the baking quality (46). However, breeding strategies for quality are different than those for yield and, for some traits, even opposite. This could be explained by knowing that the major component in the wheat grain is starch, accounting for approximately 70% of the grain’s dry weight, so increasing the yield means increasing grain starch, which implies a dilution in other grain components, including protein (47), and hence the negative correlation we found in our study between flour protein content and wheat yield (Table 12).

In accordance with the literature, drought stress can deeply influence wheat protein (31), due to its influence in plant water and (so) chlorophyll content, photosynthesis efficiency, or growth inhibition (48, 49). In our study, the main quality traits of wheat grain were studied, determining the influence of the Environment on them. Here, it outstood the negative correlation found between rainfall and protein content, as well as the positive correlation between some quality traits such as wet gluten or W with grain/flour protein content (Table 12), already referred to in the literature (50, 51). This finding could explain why, in Environment 14, with less than 70 mm rainfall in winter and 90 mm rainfall in spring, it is considered as dry environment (Supplementary Figure S14) and shows statistically the best results in wheat quality traits and one of the worst in grain yield. However, differences in behavior between cultivars were found: Valbona, considered as new cultivar, registered the best data for protein content of the flour as well as for W and wet gluten content (Tables 6, 8, 10, respectively) in the majority of the environments. This fact was supported by PCA, which defined Valbona with these three characteristics with quite high intensity (Figure 2). The following cultivar showing the best quality traits in most of the environments was Antequera, registering the best values of W in 13 out of the 18 environments studied and being the best cultivar regarding protein of the flour or wet gluten content in 8 out of the 18 environments (most of the time without any statistical differences with Valbona).

Godara et al. (52) found, thanks to PCA, that the climate is defined mainly by rainfall and maximum temperatures. In our case, PCA revealed the great influence of spring or winter rainfall, as well as days above 30°C, to define and separate the different environments (Figures 1, 3). However, in our case, the explanation of both axes did not reach 80%, while Godara et al. (52) stated an explanation above 95%. This fact was expected because the Mediterranean climate is characterized by the erratic climate conditions. In our study, high temperatures (days with temperatures above 30°C) define environments classified as dry, such as Environments 8, 14, and 17. For their part, rainy environments such as 13 or 15 were very close to spring rainfall and yield parameters in the figures, supporting a high correlation between these last environments and the rainfall in the most important part of the wheat cycle regarding the yield. This finding agreed with the results obtained by Nayana et al. (53) who stated the reliability of PCA-based models to predict wheat yield knowing the climatic parameters of the season. The differences between cultivars regarding their response to climatic variation are leading us to widen our point of view. Breeding nowadays is not just about getting a higher yield or better quality, but getting more resilient cultivars, which can provide more stable productions. In our study, with cultivars representing new and quite old cultivars and a wide range of germplasm interesting for the Mediterranean area, Antequera cultivar showed to be the most resilient cultivar regarding climate variation, being among the more productive cultivars, as well as one of the cultivars showing the best quality traits, in very different climatic conditions. According to McGrail and McNear (54), this could be due to a modification in the root system, which can increase the rhizosphere in this way, water availability, nutrient use, and C-sequestration, which can potentially improve crop resilience. In addition, the capability of the cultivar to lengthen or shorten their cycle according to weather should also be a good aim to achieve in breeding programs, because some level of earliness could be identified as a very important tool to escape the water scarcity period (55).

## 5 Concluding remarks

Today, plant breeders are facing different challenges in cereal improvement, such as changing diets, food safety, requirements in developing countries, etc., but one of the most important challenges that plant breeders are trying to achieve is the adaptation of the wheat cultivars to climate change. The studied cultivars represent the genetic advances in the last 20 years, with yields ranging between 3,000 and 9,000 kg ha^−1^ in any type of environment and registering never less than 11% of protein in the flour. They could be clear examples of quite resilient cultivars, meeting the requirements of yield (Paiva and Antequera) and dough quality (Valbona and Antequera) in many different Mediterranean climatic conditions. In conclusion, regarding yield and technological quality (Valbona, Paiva, but especially Antequera), this could be a good starting point for breeders in the process of definiting the ideotype of wheat plants for Mediterranean regions.

## Data availability statement

The raw data supporting the conclusions of this article will be made available by the authors, without undue reservation.

## Author contributions

BM: Conceptualization, Funding acquisition, Investigation, Project administration, Supervision, Writing – review & editing. RC: Data curation, Investigation, Methodology, Supervision, Writing – original draft, Writing – review & editing. CG: Conceptualization, Data curation, Investigation, Methodology, Writing – review & editing. AB: Data curation, Investigation, Methodology, Visualization, Writing – review & editing. NP: Investigation, Methodology, Writing – review & editing. JM: Data curation, Investigation, Methodology, Writing – review & editing. AC: Data curation, Investigation, Methodology, Writing – review & editing. MP: Data curation, Methodology, Validation, Writing – review & editing. JD: Data curation, Investigation, Methodology, Writing – review & editing. SR: Data curation, Formal analysis, Supervision, Validation, Visualization, Writing – original draft, Writing – review & editing.

## Footnotes

^1^ https://www.ipcc.ch/2021/08/09/ar6-wg1-20210809-pr/

^2^ https://www.fao.org/soils-portal/data-hub/soil-classification/en/
