# Supplementary material for: Breeding in bread-making wheat varieties for Mediterranean climate: the need to get resilient varieties
Source: Front Nutr. 2024 Aug 7;11:1393076. doi: 10.3389/fnut.2024.1393076 (PMC11335553; doi:10.3389/fnut.2024.1393076)
Supplement: Supplementary file 1 [file Table_1.docx]

Supplementary Material

Table S1. Comparison between treatments cultivars and environments. Sum of squares and signification level (* p ≤ 0.05; ** p ≤ 0.01; *** p ≤ 0.001) of the two-way ANOVA, including in the model ‘cultivar’, ‘environment’ and their interaction on each parameter evaluated: P, L and crude protein in the grain. DF: degree of freedom; SS: Sum of squares;

| **Source** | **P (mm)** | | **L (mm)** | | **Crude Protein in grain (%)** | |
| --- | --- | --- | --- | --- | --- | --- |
|  | **DF** | **SS** | **DF** | **SS** | **DF** | **SS** |
| Cultivar (C) | 3 | 19643.60*** | 3 | 16425.53*** | 3 | 61.78*** |
| Environment (E) | 17 | 6488.77*** | 17 | 52049.44*** | 17 | 210.71*** |
| C x E | 51 | 9831.82*** | 51 | 17969.29** | 51 | 54.87*** |

Table S2. Tenacity or P (mm) results affected by the interaction Cultivar*Environment. Averages in the same row or the same column, with different lowercase letters mean significant effect of Variety or Environment (p ≤ 0.05) respectively according to LSD test. Uppercase letters mean significant effect of Cultivar or Environment (p ≤ 0.05) respectively according to LSD test for main factors.

|  | P (mm) | | | | |
| --- | --- | --- | --- | --- | --- |
|  | Antequera | Paiva | Roxo | Valbona | Average |
| **Common**  Env. 2  Env. 3  Env. 6  Env. 9  Env. 12  **Rainy**  Env. 4  Env. 5  Env. 13  Env. 15  Env. 16  **Dry**  Env.1  Env. 8  Env. 10  Env. 11  Env. 14  Env. 17  **Special**  Env. 18  Env. 7  **AVERAGE** | 96.40±0.85 b-h  82.65±4.74 i-o  87.00±9.90 g-n  80.83±0.01 j-p  101.30±2.40 b-f  94.67±8.01 d-i  78.50±2.12 k-q  88.00±4.24 g-m  89.87±5.84 f-l  93.00±0.01 e-j  76.80±5.80 m-s  96.50±3.54 b-h  75.60±10.18 m-s  81.10±10.04 j-p  91.00±12.73 f-k  99.50±6.36 b-g  80.83±0.01 j-p  97.10±4.38 b-h  88.37± 1.58B | 61.75±17.32 v-aa  77.50±3.54 l-q  60.17±4.48 w-aa  72.00±18.38 o-w  94.45±0.78 d-i  69.50±2.12 p-x  69.50±0.71 p-x  73.00±4.24 o-v  69.50±0.71 p-x  79.00±2.83 k-q  61.45±1.77 v-aa  90.50±6.36 f-k  56.35±9.97 y-aa  95.60±4.10 b-h  75.50±7.78 m-s  79.15±1.20 k-q  77.25± 0.35 l-r  70.70±4.67 o-x  74.05±2.06 C | 74.90±1.27 n-u  52.95±1.34 z-aa  59.84±4.48 w-aa  64.00±2.83 s-z  79.55±2.19 k-q  75.25±0.35 m-t  53.00±1.41 z-aa  58.00±0.01 x-aa  62.50±7.78 t-aa  76.40±5.94 m-s  51.10±2.83 aa  64.50±0.71 r-z  61.80±6.51 v-aa  62.25±7.42 u-aa  60.50±2.12 v-aa  67.00±1.41 q-y  60.75±1.06 v-aa  96.13±12.55 b-h  65.58±1.95 D | 105.45±3.46 a-e  80.83±0.01 j-p  106.50±9.19 a-d  78.50±13.44 k-q  80.83±0.01 j-p  87.50±3.54 g-n  86.00±4.24 h-n  78.50±3.54 k-q  95.00±8.49 c-i  117.00±9.90 a  99.40±9.19 b-g  107.00±5.66 a-d  108.00±12.45 a-b  96.90±8.34 b-h  107.50±7.78 a-c  107.00±2.83 a-d  77.50±0.71 l-q  96.30±2.40 b-h  95.32±2.29 A | 84.63±7.41 B-D  73.48±4.92 F-H  78.38±8.21 D-G  73.83±4.21 F-H  89.03±3.74 A-C  81.73±4.21 DE  71.75±5.02 H  74.38±4.50 F-H  79.22±5.79 D-F  91.35±6.72 A  72.19±7.52 GH  89.63±6.46 A-C  75.44±8.60 E-H  83.96±6.07 B-D  83.63±7.47 CD  88.16±6.51 A-C  74.08±7.42 F-H  90.06±4.96 AB  80.83±1.38 |

Table S3. Extensibility or L (mm) results affected by the interaction Cultivar*Environment. Averages in the same row or the same column, with different lowercase letters mean significant effect of Variety or Environment (p ≤ 0.05) respectively according to LSD test. Uppercase letters mean significant effect of Cultivar or Environment (p ≤ 0.05) respectively according to LSD test for main factors.

|  | L (mm) | | | | |
| --- | --- | --- | --- | --- | --- |
|  | Antequera | Paiva | Roxo | Valbona | Average |
| **Common**  Env. 2  Env. 3  Env. 6  Env. 9  Env. 12  **Rainy**  Env. 4  Env. 5  Env. 13  Env. 15  Env. 16  **Dry**  Env.1  Env. 8  Env. 10  Env. 11  Env. 14  Env. 17  **Special**  Env. 18  Env. 7  **AVERAGE** | 107.65±3.75 r-z  122.25±1.77 m-v  93.84±5.42 w-aa  129.08±0.01 i-t  137.95±7.00 e-p  99.17±13.91 u-aa  125.00±7.07 j-v  151.50±0.71 a-k  107.33±0.95 r-z  148.00±1.41 b-m  147.65±14.78 c-m  132.00±2.83 i-s  160.15±31.32 a-h  124.45±10.68 k-v  168.50±33.23 a-d  122.50±3.54 l-v  129.08±0.01 i-t  97.95±4.32 v-aa  128.00±3.91 C | 89.45±7.85 y-ab  118.10±7.21 o-x  126.00±24.04 j-u  131.50±3.54 i-s  129.45±0.64 i-t  113.00±0.01 p-y  134.00±9.90 h-r  169.50±17.68 a-d  136.50±0.71 g-q  142.50±12.02 d-o  111.25±7.85 p-z  129.00±2.83 i-t  161.45±37.83 a-g  116.35±11.38 o-y  172.50±4.95 a-c  143.00±4.24 d-o  166.25±24.40 a-d  130.20±23.76 i-t  134.44±4.12 B | 108.40±13.58 r-z  137.65±7.57 e-p  116.34±0.94 o-y  164.00±31.11 a-f  119.90±5.80 n-x  122.00±2.83 m-v  149.50±7.78 b-l  164.50±12.02 a-e  127.00±9.90 i-t  136.45±3.04 g-q  136.20±12.59 g-q  173.00±12.73 a-c  174.90±37.19 a-b  125.05±16.90 j-v  178.00±8.49 a  152.00±1.41 a-j  154.00±19.80 a-i  106.58±0.81 s-z  141.41±4.27 A | 75.30±6.65 aa-ab  129.08±0.01 i-t  63.00±8.49 ab  105.50±2.12 s-z  129.08±0.01 i-t  123.50±3.54 l-v  107.00±12.73 r-z  153.50±0.71 a-i  93.00±9.90 x-aa  120.50±0.71 n-w  103.10±15.98 t-z  110.50±9.19 q-z  137.10±29.84 f-q  108.65±3.32 r-z  129.50±3.54 i-t  105.25±4.60 s-z  145.25±25.81 d-n  85.25±10.25 z-ab  112.45±4.20 D | 95.20±6.10 H  126.77±3.35 C-F  99.79±10.48 H  132.52±9.53 C  129.09±2.89 C-E  114.42±4.43 FG  128.88±6.79 C-E  159.75±4.30 A  115.96±7.13 E-G  136.86±4.53 BC  124.55±8.21 B-F  136.13±9.51 BC  158.40±11.21 A  118.63±4.29 D-F  162.13±9.20 A  130.69±7.41 CD  148.64±7.98 AB  104.99±7.62 GH  129.08±2.21 |

Table S4. Crude protein of the grain (%) results affected by the interaction Cultivar*Environment. Averages in the same row or the same column, with different lowercase letters mean significant effect of Variety or Environment (p ≤ 0.05) respectively according to LSD test

|  | Crude protein (grain) (%) | | | | |
| --- | --- | --- | --- | --- | --- |
|  | Antequera | Paiva | Roxo | Valbona | Average |
| **Common**  Env. 2  Env. 3  Env. 6  Env. 9  Env. 12  **Rainy**  Env. 4  Env. 5  Env. 13  Env. 15  Env. 16  **Dry**  Env.1  Env. 8  Env. 10  Env. 11  Env. 14  Env. 17  **Special**  Env. 18  Env. 7  **AVERAGE** | 13.35±0.49 z-ab  14.00±0.07 u-aa  14.10±0.71 t-aa  15.39±0.01 l-s  16.16±0.08 h-o  12.55±0.21 ab-ac  14.55±0.07 r-z  15.35±0.35 l-s  14.80±0.28 p-x  16.10±0.01 i-o  16.01±1.54 i-p  15.45±0.07 k-s  17.03±0.95 d-j  15.15±0.35 n-v  17.45±0.07 c-g  17.70±0.57 c-e  15.39±0.01 l-s  13.35±0.05 z-ab  15.22±0.24 C | 12.05±0.64 ac  13.61±0.42 x-ab  13.50±0.42 y-ab  13.94±0.08 v-aa  15.26±0.35 l-t  12.54±0.06 ab-ac  14.35±0.21 r-z  15.00±0.42 o-w  14.55±0.64 r-z  15.85±0.08 j-q  15.40±0.71 l-s  14.50±0.28 r-z  13.98±2.29 u-aa  15.00±0.99 o-w  16.00±0.57 i-p  16.25±0.07 g-n  16.40±0.29 f-m  12.91±0.01 aa-ac  14.50±0.23 D | 14.45±0.21 r-z  14.43±0.11 r-z  14.50±0.28 r-z  15.18±0.11 m-u  16.34±0.05 g-n  13.78±0.17 w-aa  15.00±0.28 o-w  15.35±0.07 l-s  14.70±0.14 q-y  16.19±0.13 h-o  17.00±0.01 d-j  15.15±0.35 n-v  16.45±1.34 f-l  17.15±0.92 c-i  17.15±0.07 c-i  17.10±0.28 d-i  17.33±0.25 c-h  13.04±0.76 aa-ac  15.57±0.22 B | 14.30±1.13 s-z  15.39±0..01 l-s  15.15±0.64 n-v  14.41±0.30 r-z  15.39±0.01 l-s  15.55±0.21 k-r  15.95±0.49 i-p  17.95±0.21 b-d  15.50±0.28 k-s  17.59±0.12 c-f  16.30±1.56 g-n  16.10±0.71 i-o  19.45±2.05 a  16.65±0.35 e-k  18.95±0.64 ab  16.30±0.01 g-n  18.37±0.04 a-c  13.86±0.08 w-aa  16.29±0.28 A | 13.54±0.44 J  14.36±0.28 HI  14.31±0.29 I  14.73±0.24 G-I  15.79±0.20 EF  13.60±0.50 J  14.96±0.26 GH  15.91±0.49 DE  14.89±0.18 G-I  16.43±0.28 B-D  16.18±0.40 C-DE  15.30±0.26 FG  16.73±0.93 BC  15.99±0.43 DE  17.39±0.44 A  16.84±0.26 AB  16.87±0.45 AB  13.29±0.18 J  15.40±0.13 |
