# Supplementary material for: Breeding in bread-making wheat varieties for Mediterranean climate: the need to get resilient varieties
Source: Front Nutr. 2024 Aug 7;11:1393076. doi: 10.3389/fnut.2024.1393076 (PMC11335553; doi:10.3389/fnut.2024.1393076)
Supplement: Supplementary file 2 [file Image_1.pdf]

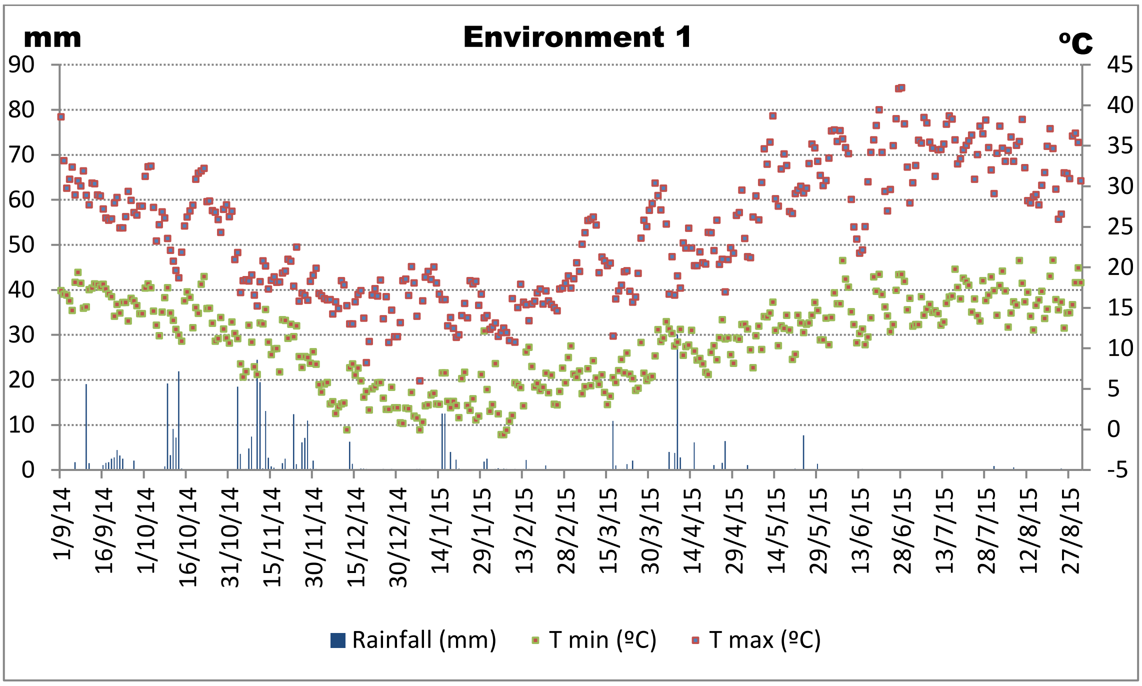

Figure S1. Termopluviometric graphic with daily rainfall and maximum and minimum temperatures in Environment 1.

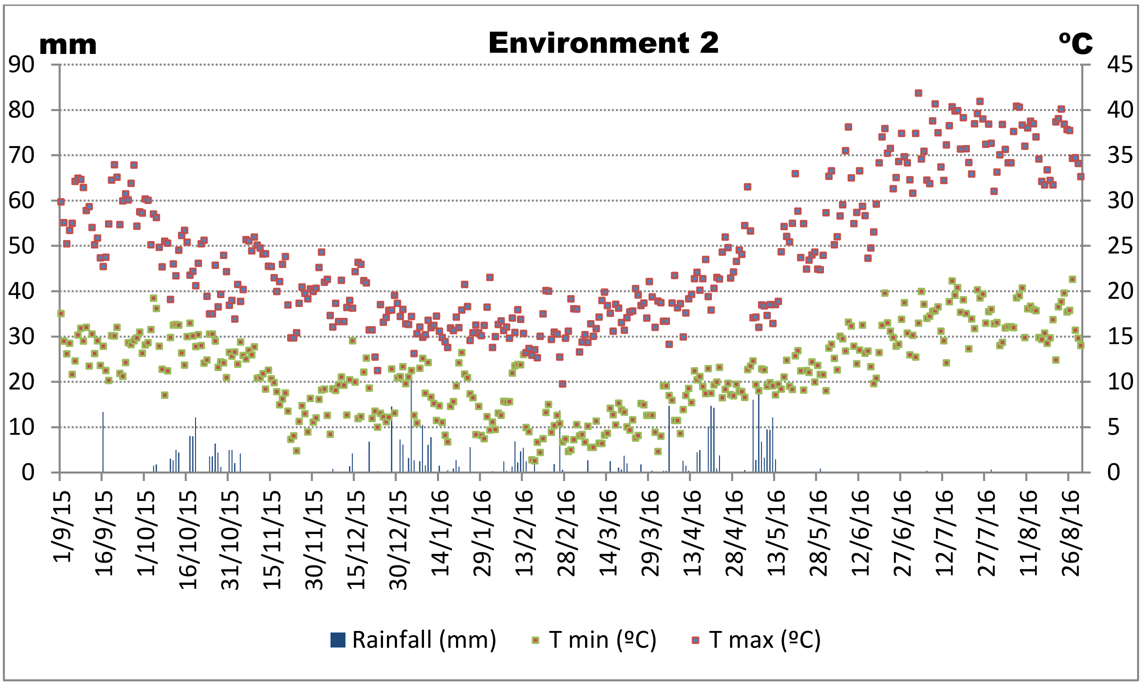

Figure S2. Termopluviometric graphic with daily rainfall and maximum and minimum temperatures in Environment 2.

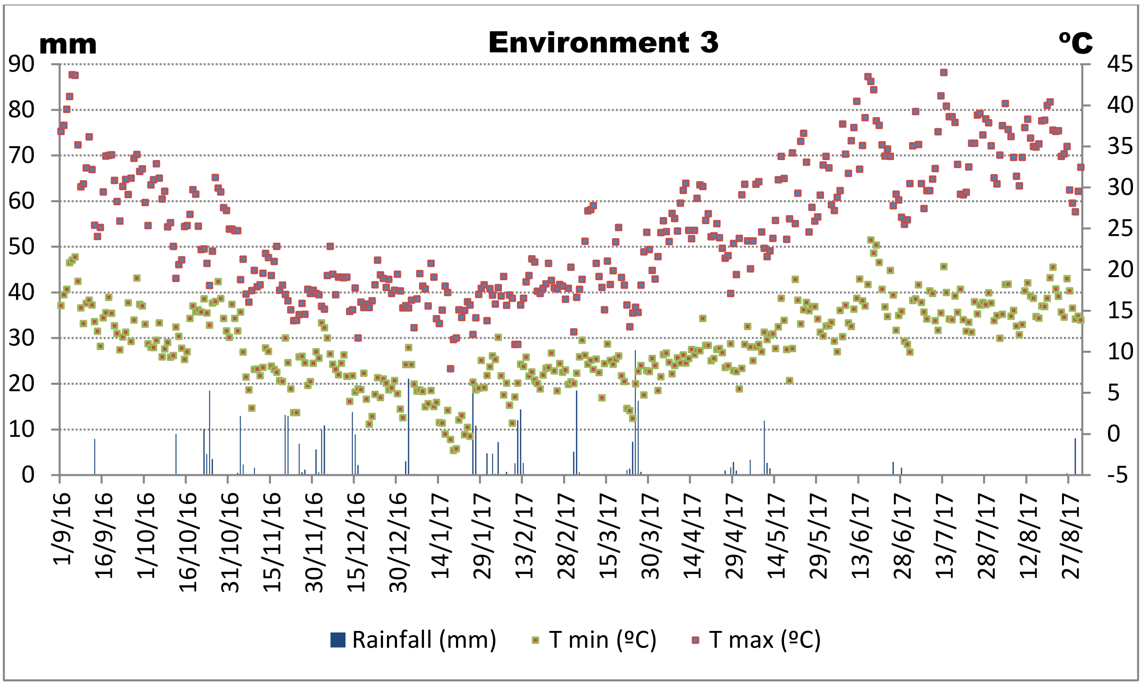

Figure S3. Termopluviometric graphic with daily rainfall and maximum and minimum temperatures in Environment 3.

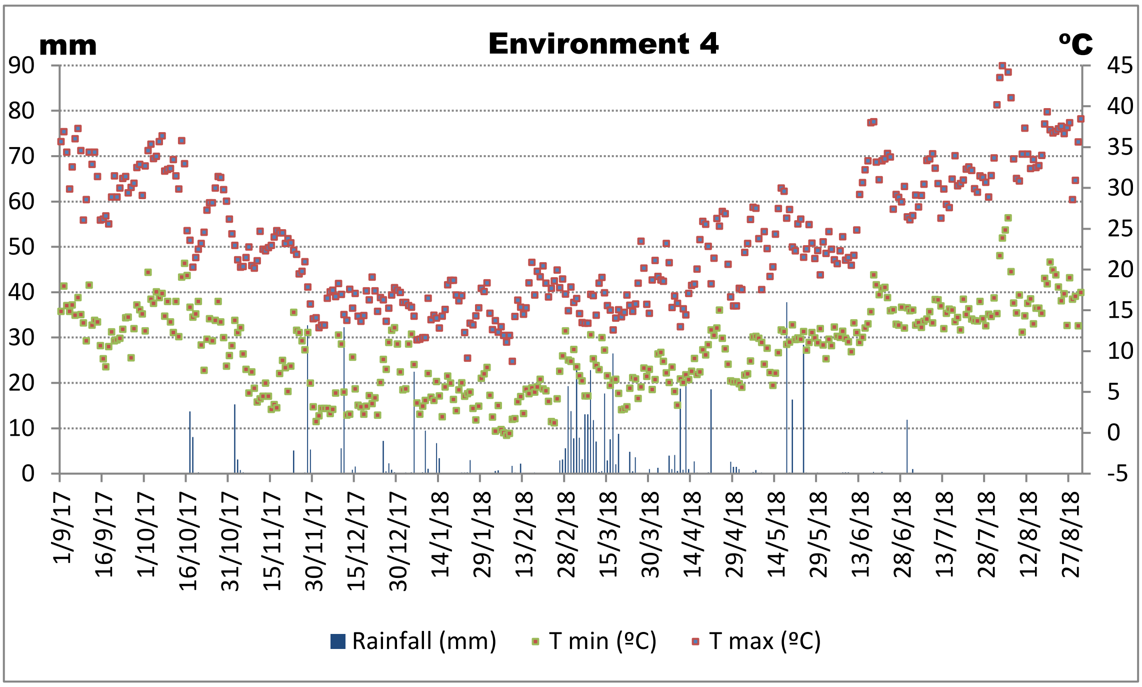

Figure S4. Termopluviometric graphic with daily rainfall and maximum and minimum temperatures in Environment 4.

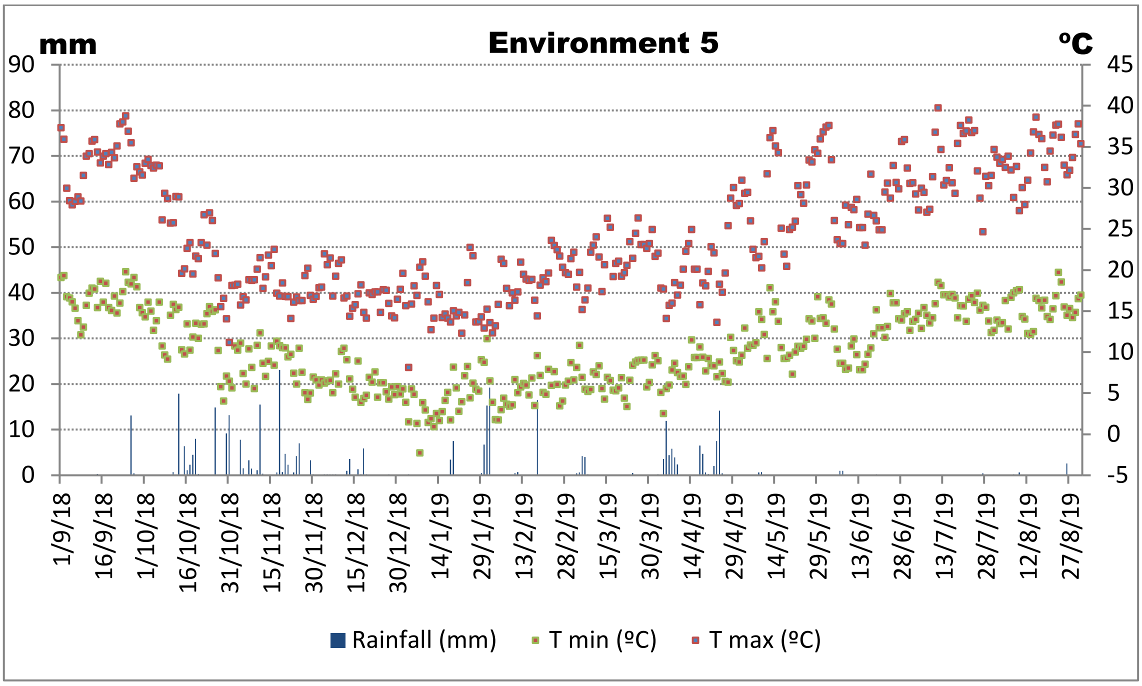

Figure S5. Termopluviometric graphic with daily rainfall and maximum and minimum temperatures in Environment 5.

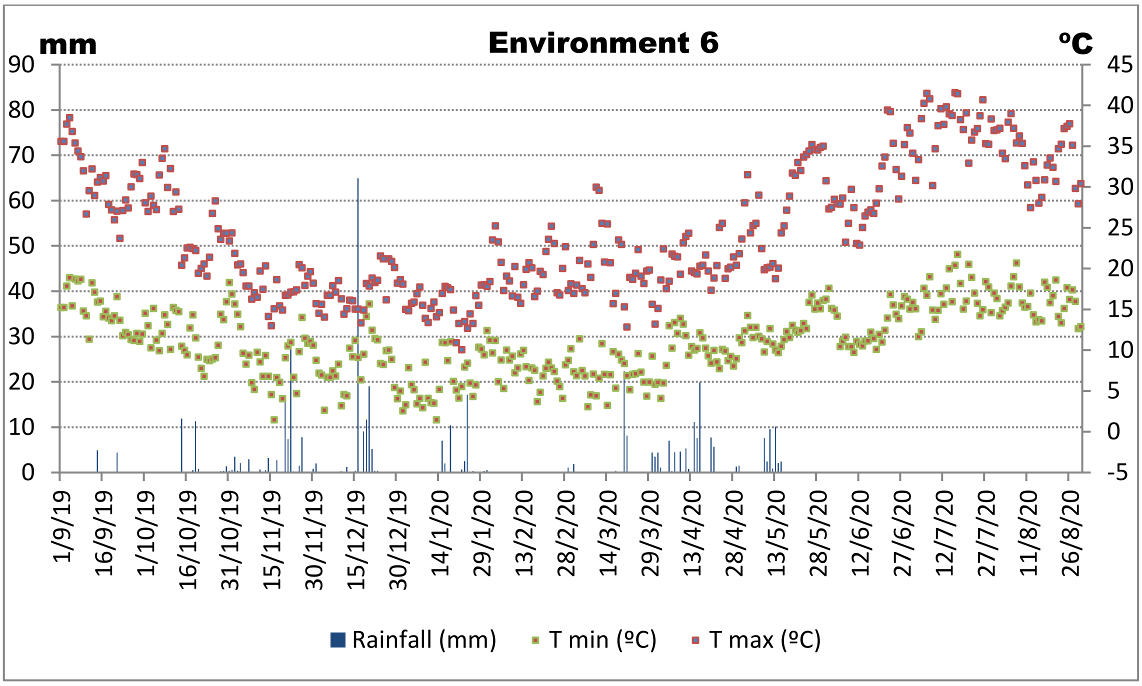

Figure S6. Termopluviometric graphic with daily rainfall and maximum and minimum temperatures in Environment 6.

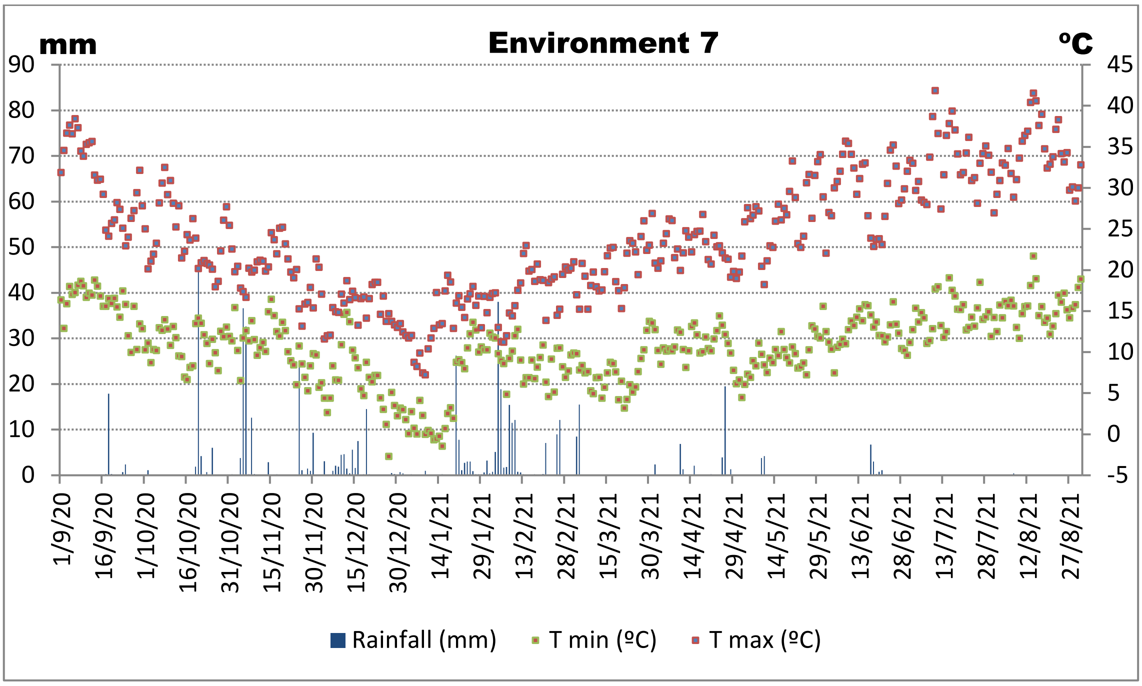

Figure S7. Termopluviometric graphic with daily rainfall and maximum and minimum temperatures in Environment 7.

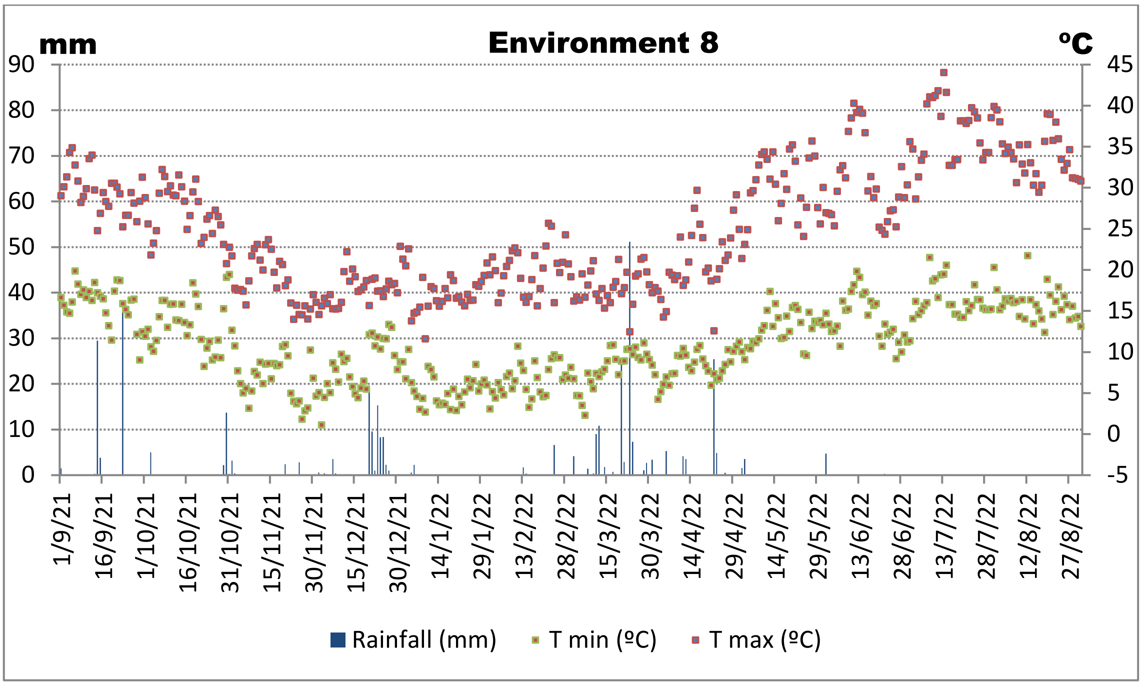

Figure S8. Termopluviometric graphic with daily rainfall and maximum and minimum temperatures in Environment 8.

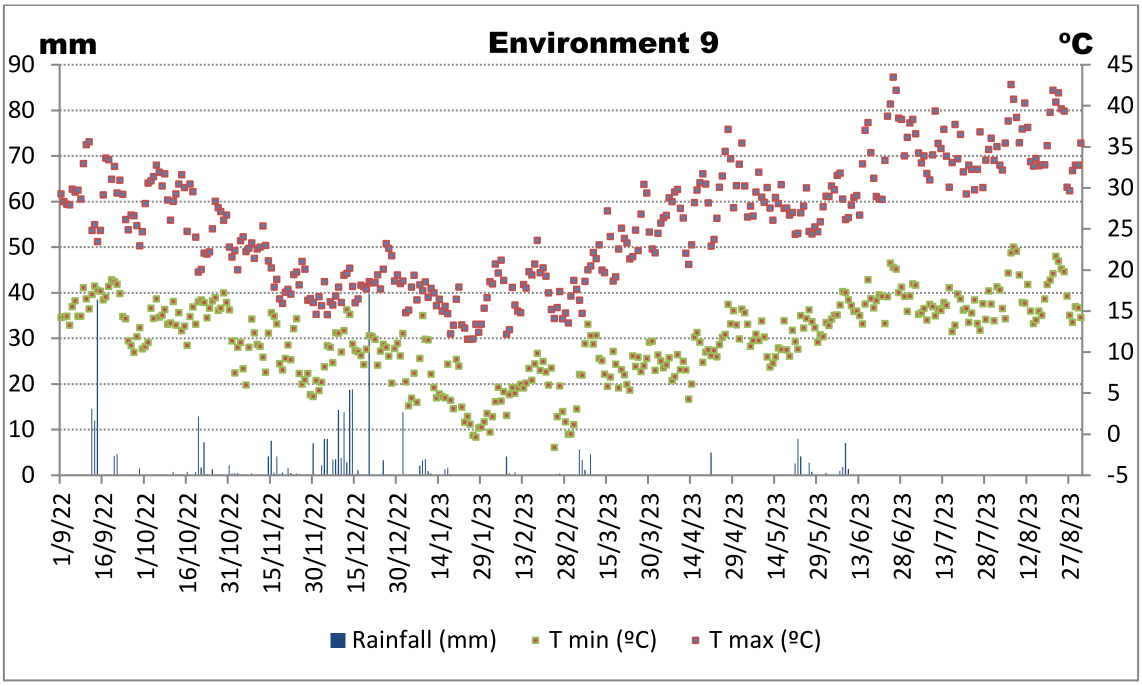

Figure S9. Termopluviometric graphic with daily rainfall and maximum and minimum temperatures in Environment 9.

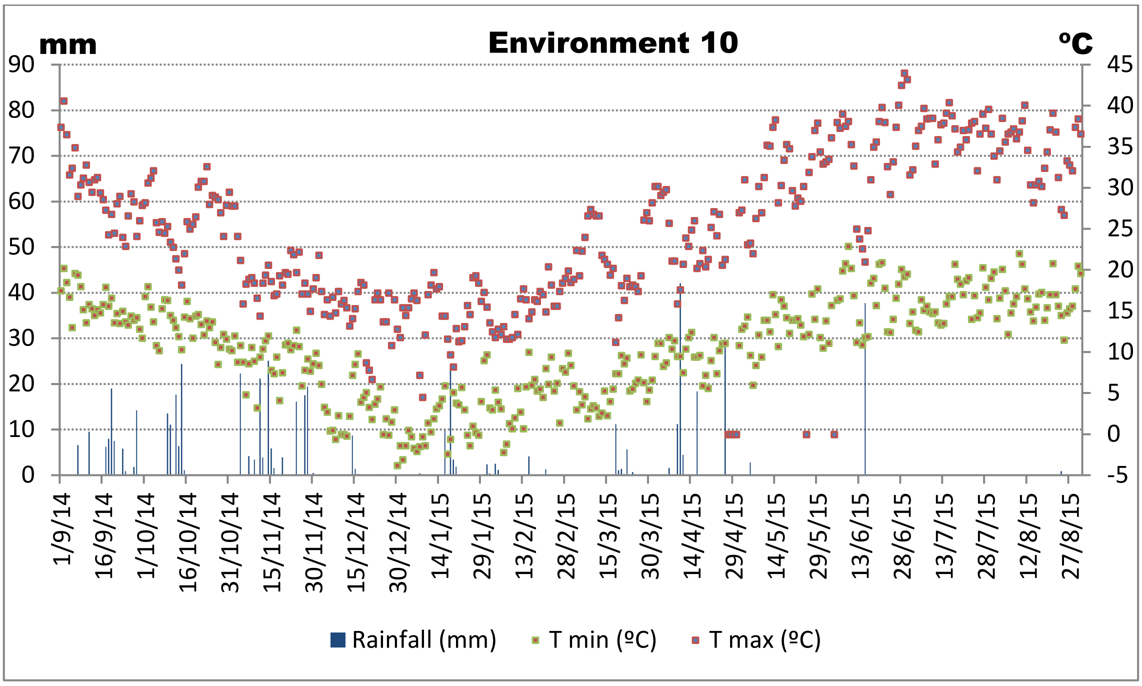

Figure S10. Termopluviometric graphic with daily rainfall and maximum and minimum temperatures in Environment 10.

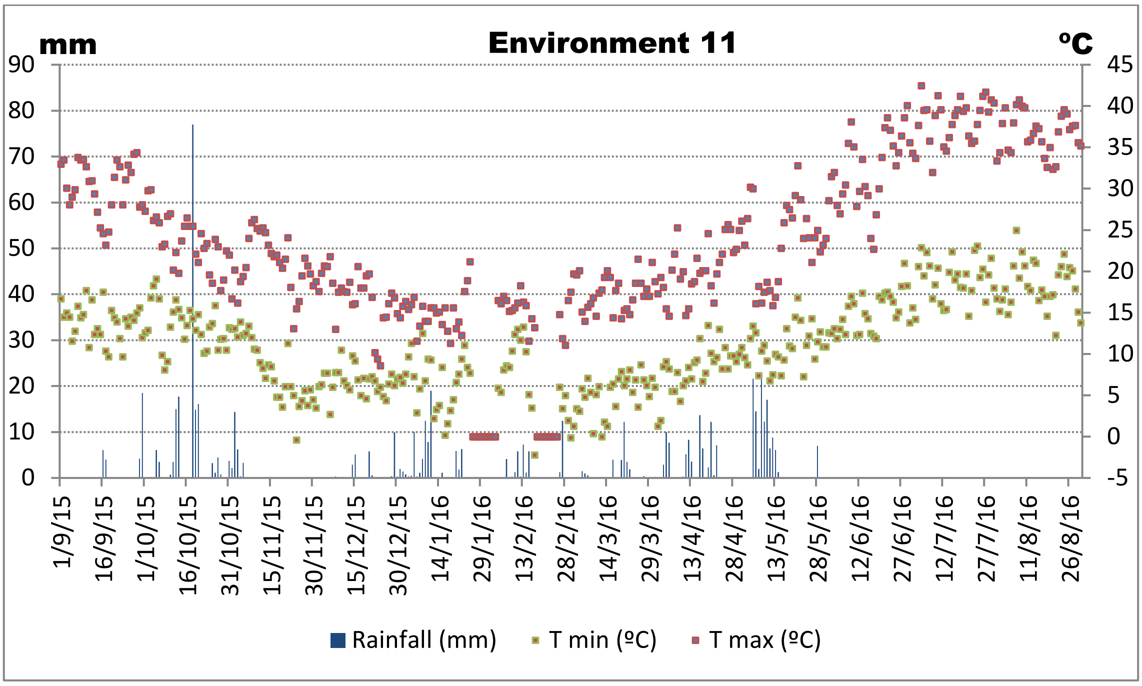

Figure S11. Termopluviometric graphic with daily rainfall and maximum and minimum temperatures in Environment 11.

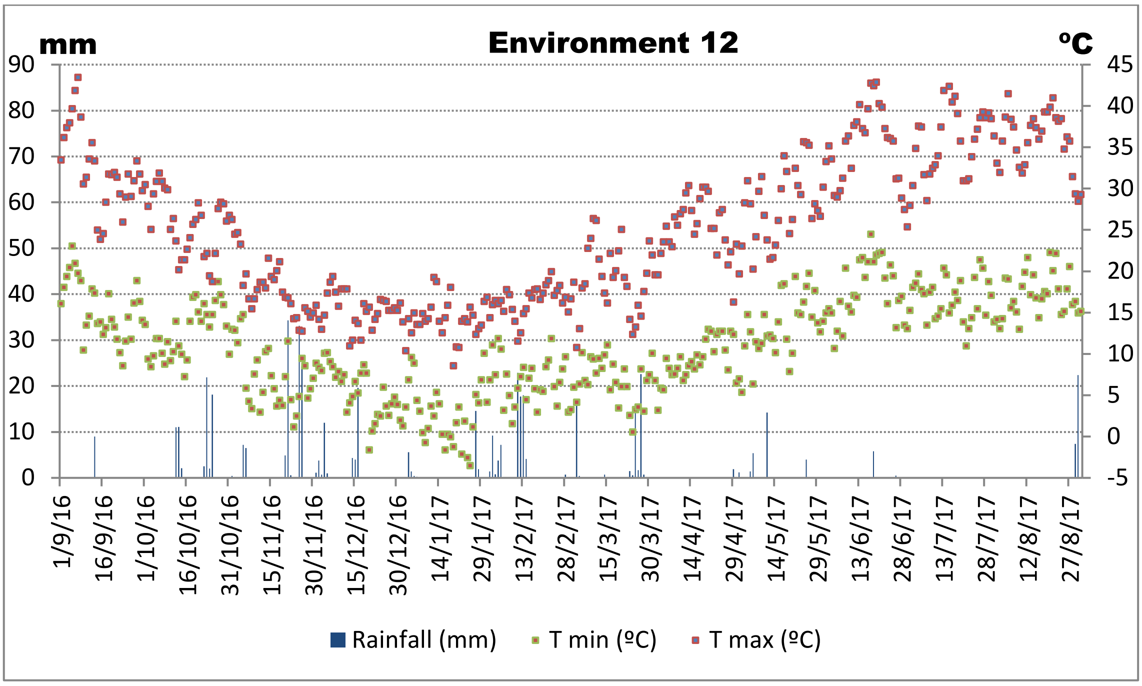

Figure S12. Termopluviometric graphic with daily rainfall and maximum and minimum temperatures in Environment 12.

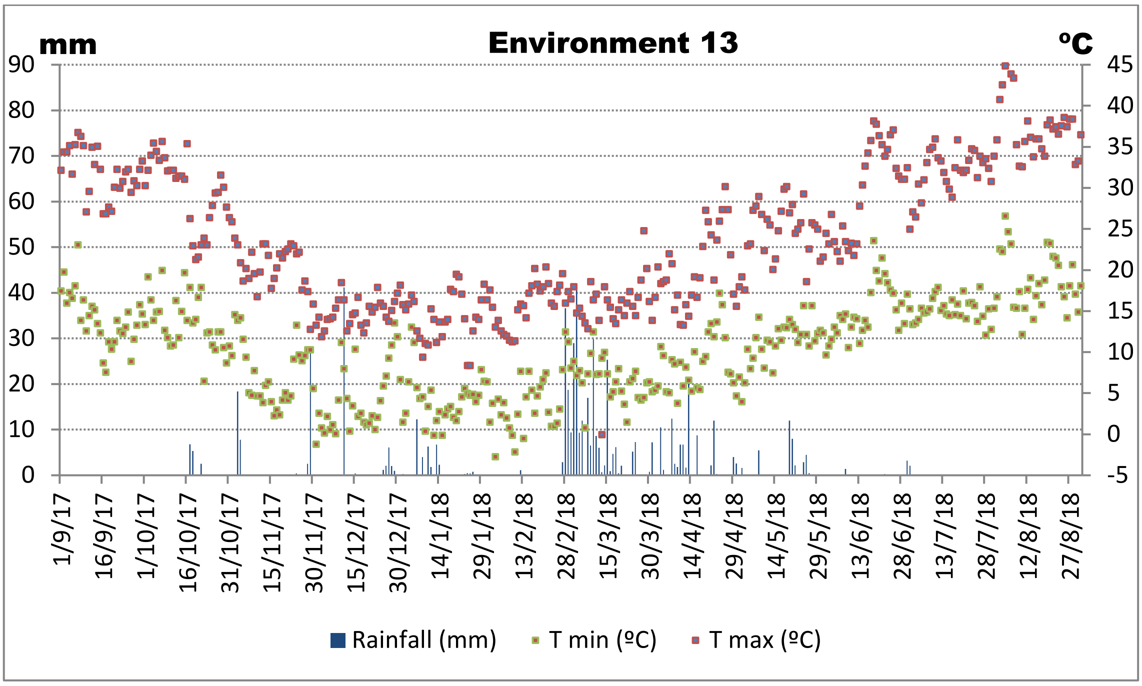

Figure S13. Termopluviometric graphic with daily rainfall and maximum and minimum temperatures in Environment 13.

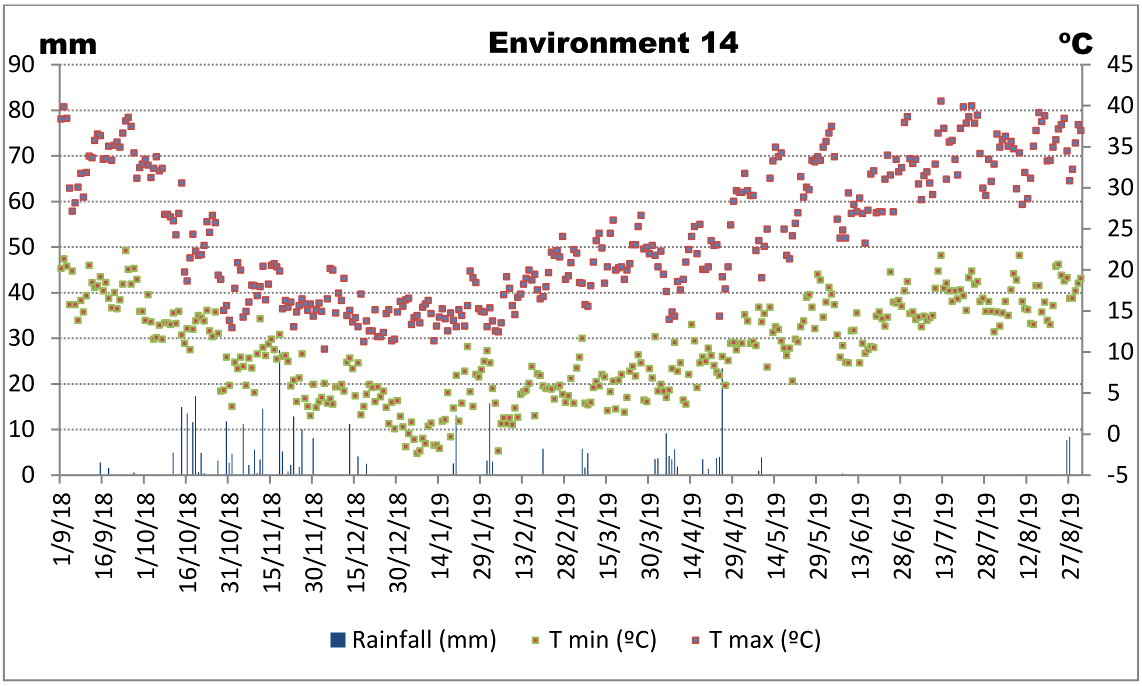

Figure S14. Termopluviometric graphic with daily rainfall and maximum and minimum temperatures in Environment 14.

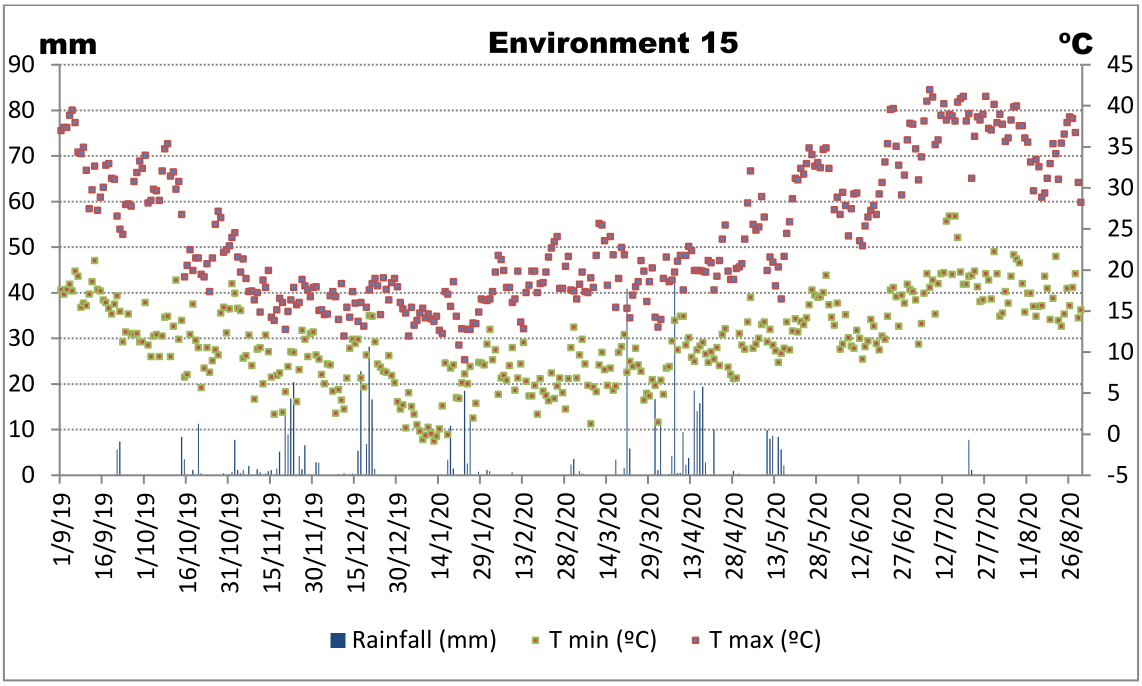

Figure S15. Termopluviometric graphic with daily rainfall and maximum and minimum temperatures in Environment 15.

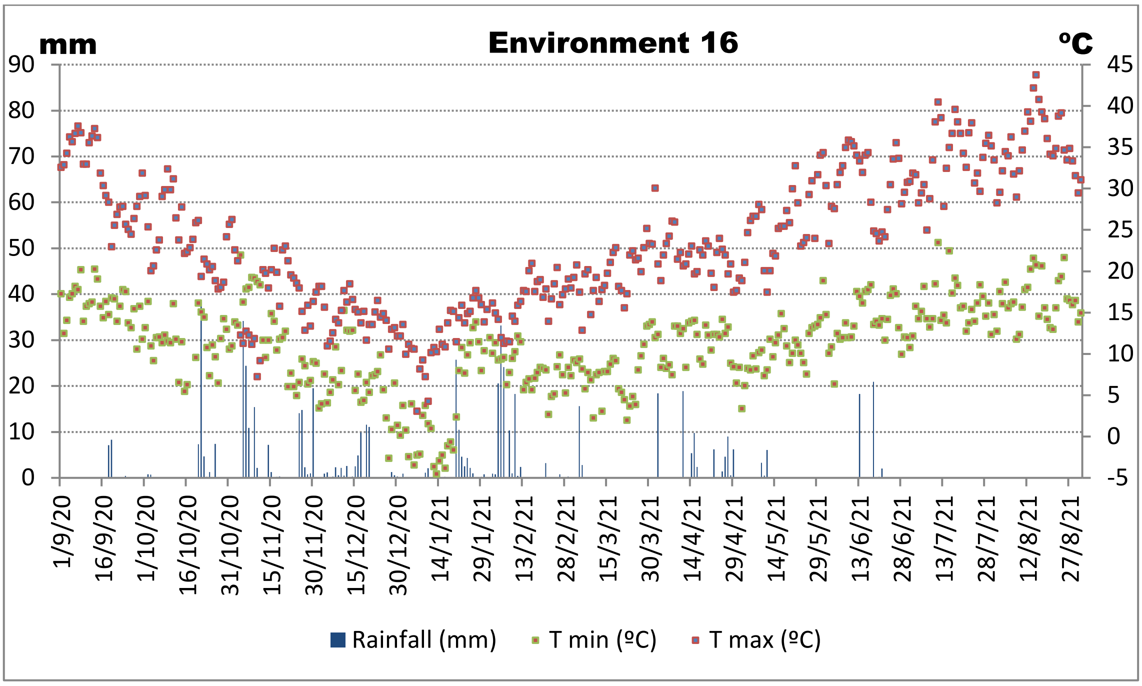

Figure S16. Termopluviometric graphic with daily rainfall and maximum and minimum temperatures in Environment 16.

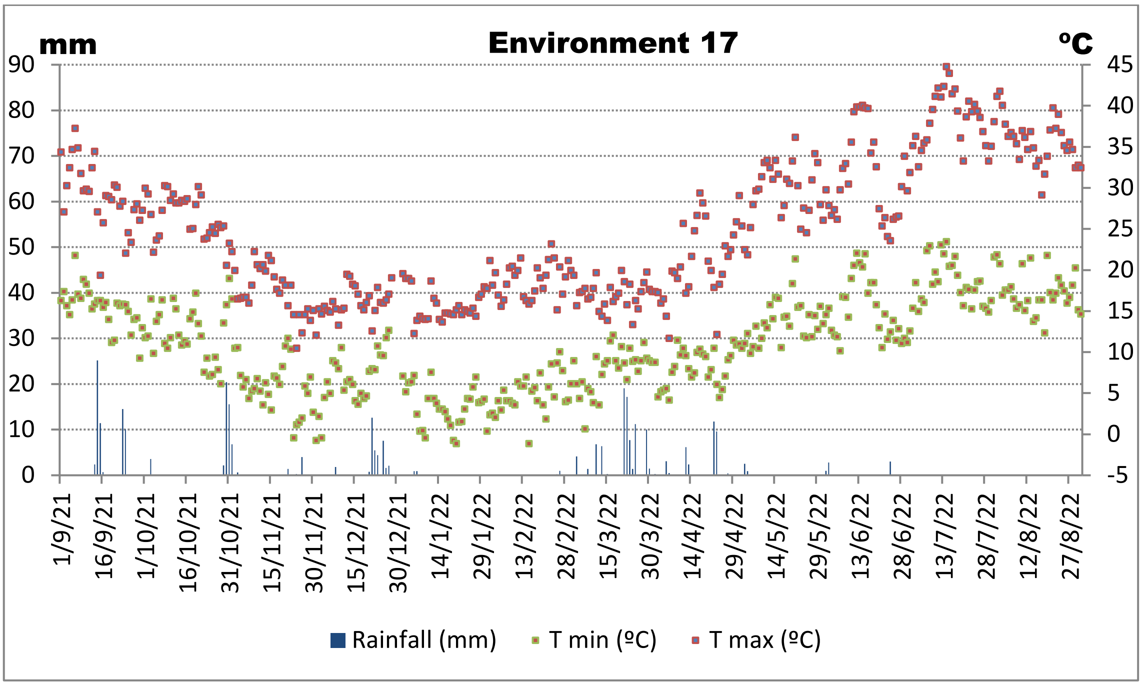

Figure S17. Termopluviometric graphic with daily rainfall and maximum and minimum temperatures in Environment 17.

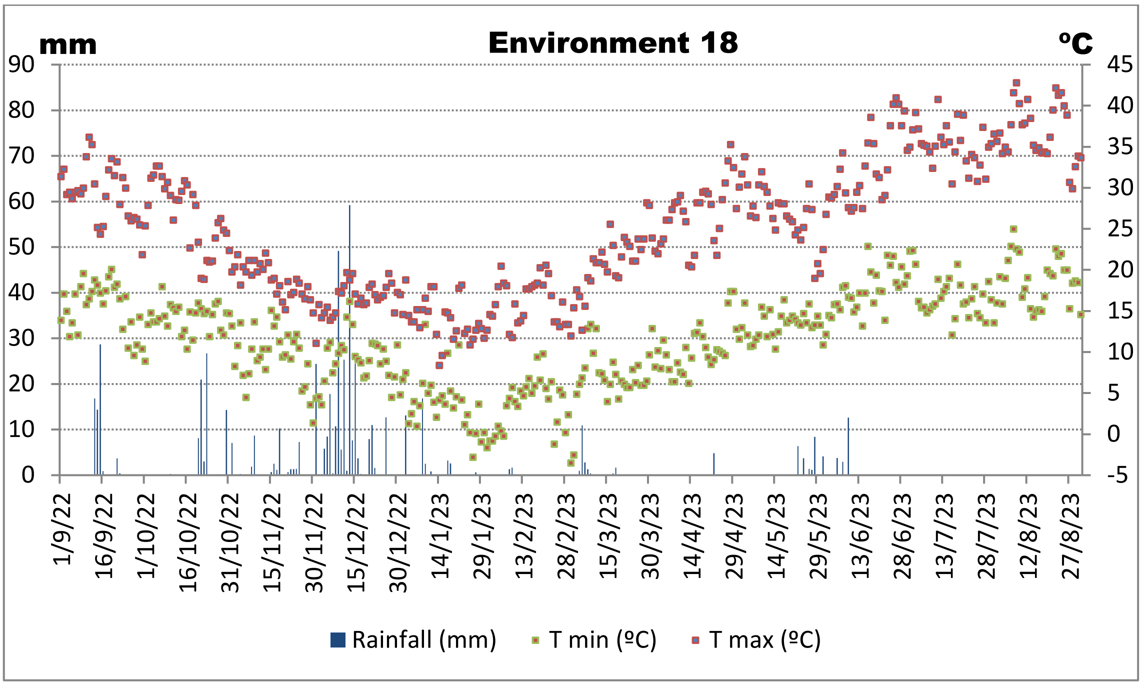

Figure S18. Termopluviometric graphic with daily rainfall and maximum and minimum temperatures in Environment 18.
